# Actin filament dynamics impacts keratinocyte stem cell maintenance

**DOI:** 10.1002/emmm.201201839

**Published:** 2013-04-02

**Authors:** Daisuke Nanba, Fujio Toki, Natsuki Matsushita, Sachi Matsushita, Shigeki Higashiyama, Yann Barrandon

**Affiliations:** 1 Laboratory of Stem Cell Dynamics, Ecole Polytechnique Fédérale de Lausanne (EPFL)Lausanne, Switzerland; 2 Department of Experimental Surgery, Centre Hospitalier Universitaire Vaudois (CHUV)Lausanne, Switzerland; 3 Senior Research Fellow Center, Ehime UniversityShitsukawa, Toon, Ehime, Japan; 4 Proteo-Medicine Research Center (ProMRes), Ehime UniversityShitsukawa, Toon, Ehime, Japan; 5 Department of Biochemistry and Molecular Genetics, Graduate School of Medicine, Ehime UniversityShitsukawa, Toon, Ehime, Japan

**Keywords:** actin filaments, clonal conversion, EGF, EGFR signalling, keratinocyte stem cells

## Abstract

Cultured human epidermal keratinocyte stem cells (holoclones) are crucial for regenerative medicine for burns and genetic disorders. In serial culture, holoclones progressively lose their proliferative capacity to become transient amplifying cells with limited growth (paraclones), a phenomenon termed clonal conversion. Although it negatively impacts the culture lifespan and the success of cell transplantation, little is known on the molecular mechanism underlying clonal conversion. Here, we show that holoclones and paraclones differ in their actin filament organization, with actin bundles distributed radially in holoclones and circumferentially in paraclones. Moreover, actin organization sets the stage for a differing response to epidermal growth factor (EGF), since EGF signalling induces a rapid expansion of colony size in holoclones and a significant reduction in paraclones. Furthermore, inhibition of PI3K or Rac1 in holoclones results in the reorganization of actin filaments in a pattern that is similar to that of paraclones. Importantly, continuous Rac1 inhibition in holoclones results in clonal conversion and reduction of growth potential. Together, our data connect loss of stem cells to EGF-induced colony dynamics governed by Rac1.

## INTRODUCTION

Actin filament organization and actomyosin contractility are critical for cell shape changes and movements during many developmental processes including gastrulation, tissue morphogenesis and remodelling (Gorfinkiel & Blanchard, 2011; Mason & Martin, 2011). The dynamics of actin filaments and bipolar assemblies of myosin II has been nicely characterized during the morphogenesis of *Caenorhabditis elegans* and *Drosophila melanogaster* embryos (Levayer & Lecuit, 2012), and in epidermal keratocyte locomotion in fish (Keren et al, 2009; Schaub et al, 2007; Small et al, 1995). In mammals, the epidermis is a superb model system to study the role of actin filament dynamics in tissue homeostasis because it constantly renews thanks to keratinocyte stem/progenitor cells located in the epithelial basal layer, and in epidermal appendages. Dividing keratinocyte stem cells generate cells with more restricted growth potential that, in turn, generate suprabasal cells that will terminally differentiate to contribute to the barrier function of the skin (Blanpain & Fuchs, 2009; Clayton et al, 2007; Jones et al, 2007; Rochat et al, 1994; Sotiropopulou & Blanpain, 2012). Moreover, actin filaments are reorganized during terminal differentiation of epidermal keratinocytes (Connelly et al, 2010; Lewis et al, 1987; Vaezi et al, 2002), through a molecular mechanism mediated by RhoA and Rac1 (Benitah et al, 2005; Vaezi et al, 2002), the small Rho GTPases that function downstream of epidermal growth factor receptor (EGFR) signalling, and other tyrosine kinase receptor pathways (Raftopoulou & Hall, 2004). However, the impact of actin filament reorganization in epidermal keratinocyte stem cells remains unknown.

**Figure 1:**
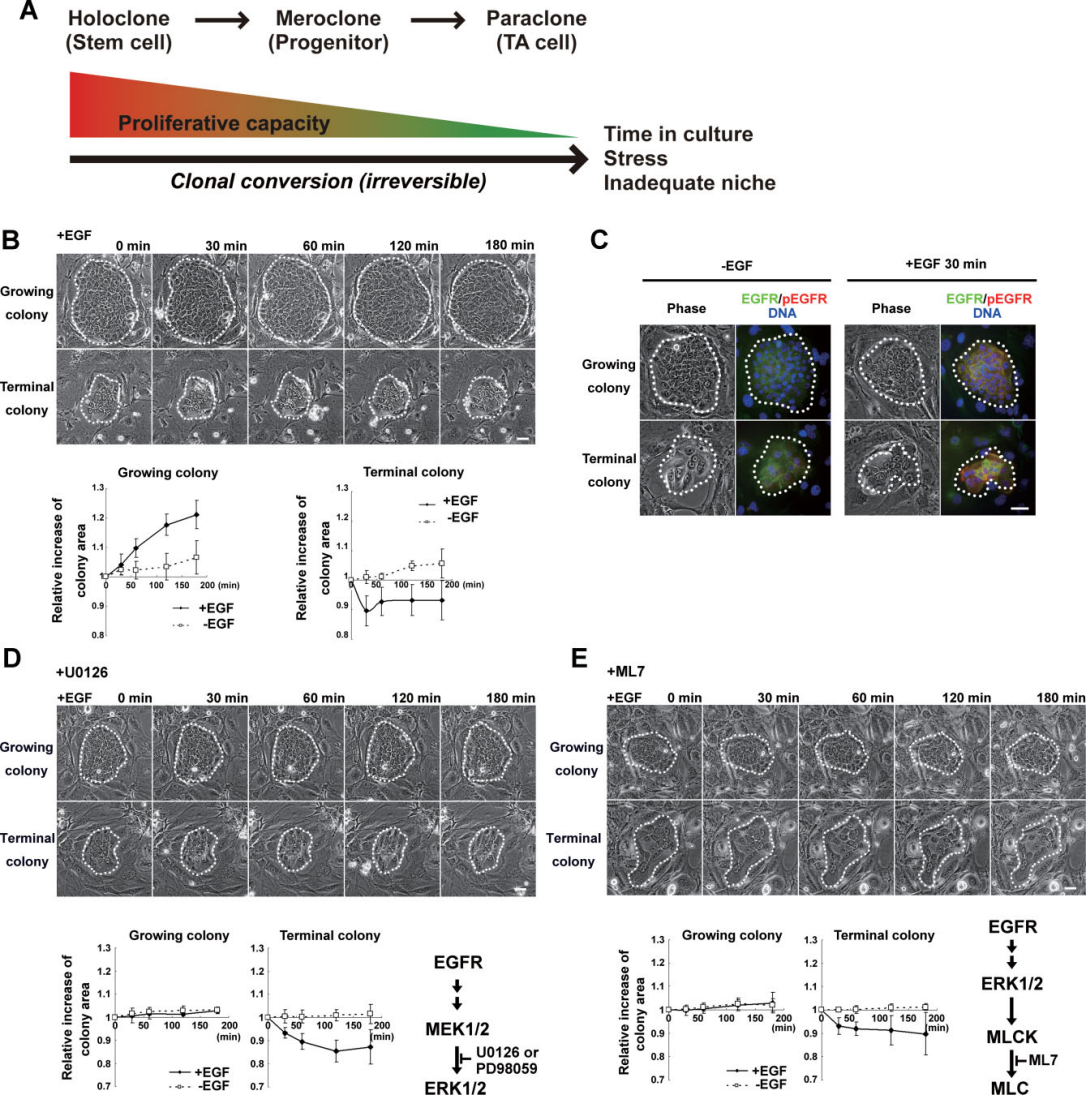
Growing and terminal human keratinocyte colonies respond differently to EGF through EGFR/ERK/MLCK signalling. A. Clonal conversion. In serial culture, a human keratinocyte stem cell (holoclone) progressively loses its proliferative capacity to become a progenitor (meroclone) and then a transient amplifying (TA) cell (paraclone) which ultimately results in stem cell loss. B. Upper panel shows phase-contrast images of growing and terminal colonies of human epidermal keratinocytes after treatment with 30 ng/ml EGF (EGF was diluted in 0.1% BSA solution). Colony edges are outlined with white dots. Lower panel shows relative increase in area of a growing and a terminal colony after EGF addition. The values (mean ± SD) were determined on results obtained from at least five colonies. BSA solution was added instead of EGF solution in no EGF condition. See also Supporting Information Figure S2F. *X*- and *Y*-axes show time (min) after addition of EGF or BSA, and relative increase in colony area (0 min = 1), respectively. Scale bar: 50 µm. C. Appearance of a growing and a terminal keratinocyte colony in phase-contrast and after immunostaining with EGFR and phosphorylated EGFR (pEGFR) antibodies. Left panel without EGF and right panel with EGF (30 ng/ml). Scale bar: 50 µm. D,E. Phase-contrast images of a growing and a terminal colony after the addition of EGF in the presence of U0126 (10 µM) (D) or ML7 (25 µM) (E). Lower panels show relative increase in colony area. Scale bar: 50 µm.

Human keratinocyte stem cells are clonogenic and can be extensively cultured (Rheinwald & Green, 1975). Under appropriate conditions, these stem cells, known as holoclones (Barrandon & Green, 1987a), can undergo at least 180 doublings, generating enough progeny to entirely reconstitute the epidermis of an adult human for a lifetime (Mathor et al, 1996; Rochat et al, 1994, 2012). Moreover, clonal analysis has demonstrated that besides stem cells, there are other clonogenic keratinocytes with restricted growth capabilities (Barrandon & Green, 1987a). First, there are progenitors (meroclones) that can only generate an epidermis for a short term when transplanted. Second, there are transient amplifying (TA) cells (paraclones), which growth capacity is limited to a maximum of 15 doublings; obviously paraclones cannot regenerate an epidermis. Termination of a culture of human keratinocytes often results from a phenomenon termed clonal conversion (Fig 1A), the switch of a holoclone into a meroclone or paraclone (Barrandon et al, 2012; Rochat et al, 2012). Clonal conversion thus results in progressive and irreversible restriction in growth potential. It is accelerated by stress, suboptimal culture conditions (inadequate niche), serial cultivation and age of donor. However, reversion of a paraclone to a stem cell-like phenotype can be obtained by immortalization or oncogenic transformation (Barrandon et al, 1989; Dellambra et al, 2000; Dürst et al, 1987). Recent results also indicate that continuous inhibition of Rho signalling (Chapman et al, 2010; McMullan et al, 2003; Terunuma et al, 2010), and continuous inhibition of mTOR signalling by rapamycin (Brouard et al., in preparation) favour the formation of progressively growing colonies while decreasing the formation of paraclones. Together, these observations suggest that clonal conversion can be reduced or even stopped. Moreover, it is essential to comprehend the molecular mechanisms that govern clonal conversion because cultured human epidermal stem cells can be transplanted onto patients with extensive burns and genetic disorders to regenerate a functional epidermis (De Luca et al, 2006; Gallico et al, 1984; Mavilio et al, 2006; Pellegrini et al, 1999; Rochat et al, 2012; Ronfard et al, 2000). Alleviating clonal conversion will improve stem cell self-renewal and engraftment, together with the long-term maintenance of the regenerated epidermis in transplanted patients.

Here, we show that colonies of keratinocyte stem cells differ from those formed by TA keratinocytes in their organization of actin filaments and in their response to EGF and that continuous Rac1 inhibition in holoclones results in clonal conversion.

## RESULTS

### Clonogenic human keratinocytes respond differently to EGF

Human diploid epidermal keratinocytes form colonies of different shapes when cultured on a feeder layer of lethally irradiated 3T3-J2 cells, at clonal density (Rheinwald & Green, 1975). After 6 days of culture, some colonies have a nearly circular shape and contain densely packed small basal cells; these colonies grow to a large size if further cultured and can be serially passaged (Supporting Information Fig S1A and S1B). Other colonies have an irregular shape and mostly contain large and flattened basal cells; these colonies soon abort growth and cannot be subcultured (Supporting Information Fig S1A and S1B). The former colonies are initiated by holoclones (stem cells) and meroclones, while the latter are initiated by paraclones (TA cells; Barrandon & Green, 1987a). Because EGFR signalling is important for the successful expansion of diploid human keratinocytes in culture (Barrandon & Green, 1987b), we have further explored how keratinocyte colonies respond to EGF. As expected, progressively growing colonies constantly increased their size during the first 120 min after addition of EGF (30 ng/ml; Barrandon & Green, 1987b), but terminal (aborted) colonies had a strikingly different behaviour. Terminal colonies rapidly shrunk before expanding again in the next 30 min, however, without fully recovering the size they had before addition of EGF (Fig 1B). Time-lapse imaging showed that the expansion of a growing colony in response to EGF was caused by two mechanisms: a centrifugal migration of the peripheral cells with maintenance of cell–cell contact, and a flattening of the cells located at the center of the colony (Supporting Information Movie S1). In contrast, reduction in size of a terminal colony resulted from the shrinking of each individual cell (Supporting Information Movie S2). This dual effect of EGF could not be explained by an altered expression and/or altered functionality of the EGF receptor, as addition of EGF induced EGFR phosphorylation and internalization in both types of colonies (Fig 1C).

EGF activates extracellular signal-regulated kinase 1 and 2 (ERK1 and ERK2), through EGFR/RAS signalling. ERK1 and ERK2 (ERK1/2) are involved in migration of various cell types (Huang et al, 2004), and epithelial cell sheets (Matsubayashi et al, 2004). As expected, ERK1/2 were immediately phosphorylated after EGF addition in human keratinocytes, and EGF-induced ERK1/2 phosphorylation was significantly decreased by U0126, a MAPK/ERK kinase (MEK) 1 and 2 inhibitor (Supporting Information Fig S2A and S2C). Interestingly, addition of U0126 completely inhibited expansion of growing colonies while affecting the dynamics of shrinking of terminal colonies (Fig 1D). Similar results were obtained with another MEK inhibitor (PD98059; Supporting Information Fig S2B). Furthermore, exposed to U0126 for more 2 days increased the expression of involucrin (INV), transglutaminase 1 (TG1), keratin 1 (K1), keratin 10 (K10) and desmoglein 1 (DSG1), and inhibited the growth of growing colonies, even when EGF was added to the culture (Supporting Information Fig S3). These data indicated that colony expansion through EGFR/ERK signalling is essential for sustained growth of keratinocyte colonies. In response to EGFR ligands, cell motility immediately increases through activation of ERK1/2 without *de novo* transcription (Huang et al, 2004). ERK1/2 then phosphorylates and activates myosin light chain kinase (MLCK; Klemke et al, 1997; Nguyen et al, 1999), which in turn phosphorylates the myosin regulatory light chain (MLC) of myosin II, resulting in actomyosin contraction and cell movement (Totsukawa et al, 2004). In keratinocytes, EGF induced MLC phosphorylation of myosin II, which decreased in presence of MEK inhibitors and a MLCK inhibitor (ML7) (Supporting Information Fig S2C and S2D). ML7 also prevented the expansion of growing colonies and resulted in a delayed shrinking of terminal colonies (Fig 1E), as MEK inhibitors did. These results demonstrated for the first time that growing and terminal colonies of keratinocytes had different dynamics in response to EGF, and indicated that their expansion and shrinking depended on MLC phosphorylation and actomyosin interaction through EGFR/ERK/MLCK signalling.

### Actin filaments organize differently in growing and terminal colonies

**Figure 2:**
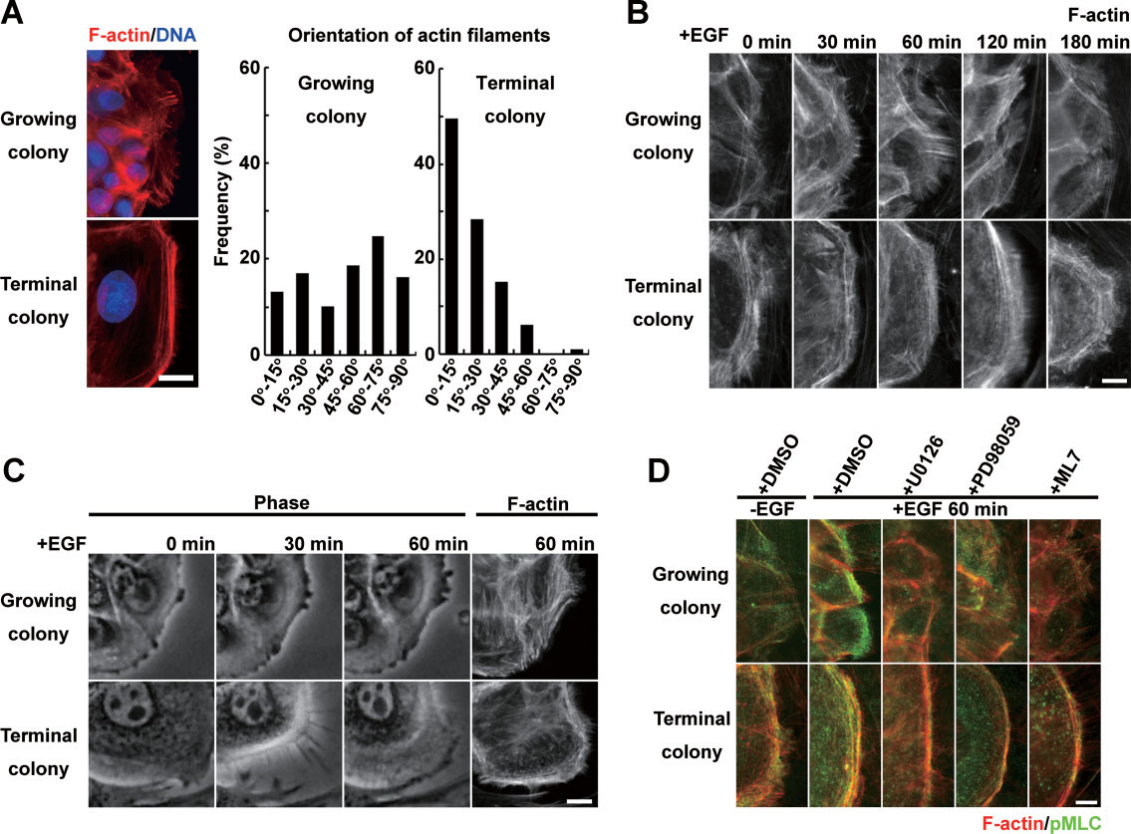
Organization of actin filaments in growing and terminal human keratinocyte colonies. A. Left panel shows rhodamine-phalloidin staining of cells localized at the periphery of a growing and a terminal colony cultured without EGF for 6 days. Right panel shows distribution of angles of actin filaments against plasma membrane in cells localized at the periphery of growing and terminal colonies without EGF. Angles of actin filaments were measured as described in Material and Methods. See also Supporting Information Figure S4. The distribution was obtained from at least 75 well-defined actin filaments in more than 25 cells of growing or terminal colonies. Scale bar: 10 µm. B. Appearance of actin filament organization in cells localized at the periphery of a growing and a terminal colony after EGF stimulation (rhodamine-phalloidin staining). Scale bar: 2.5 µm. C. First 3 panels: phase-contrast images of a cell localized at the periphery of a growing and a terminal colony after EGF stimulation. Last panels show the same cell after it was fixed and rhodamine-phalloidin stained. Scale bar: 10 µm. D. pMLC immunostaining and rhodamine-phalloidin staining of cells localized at the periphery of growing and terminal colonies after EGF stimulation and in presence of U0126 (10 µM), PD98059 (50 µM) or ML7 (25 µM). Scale bar: 2.5 µm.

Phosphorylation of MLC induces bipolar assemblies of myosin II, and enables myosins to interact with actin filaments. Hence, we next visualized the network of actin filaments in growing and terminal colonies by rhodamine-phalloidin staining. In absence of EGF, short actin bundles were distributed radially in the cells located at the periphery of growing colonies, whereas a well-developed circumferential actin network was observed in cells of terminal colonies (Fig 2A). Quantitative analysis clearly revealed that orientation of actin filaments in the cells located at the periphery of colonies was significantly different in growing and terminal colonies (Fig 2A and Supporting Information Fig S4). In response to EGF, cytoplasmic protrusions were observed together with radial actin bundles at the leading edge of cells of growing colonies but not in cells of terminal colonies (Fig 2B and C). In both colony types, phosphorylated MLC of myosin II colocalized with actin filaments and was more intense after EGF exposure, whereas it significantly decreased after exposure to MEK and MLCK inhibitors, even in the presence of EGF (Fig 2D). These data further indicated that EGF induced actomyosin interaction through EGFR/ERK/MLCK signalling in growing and terminal colonies of human keratinocytes.

### Clonal conversion results in the reorganization of actin filaments

**Figure 3:**
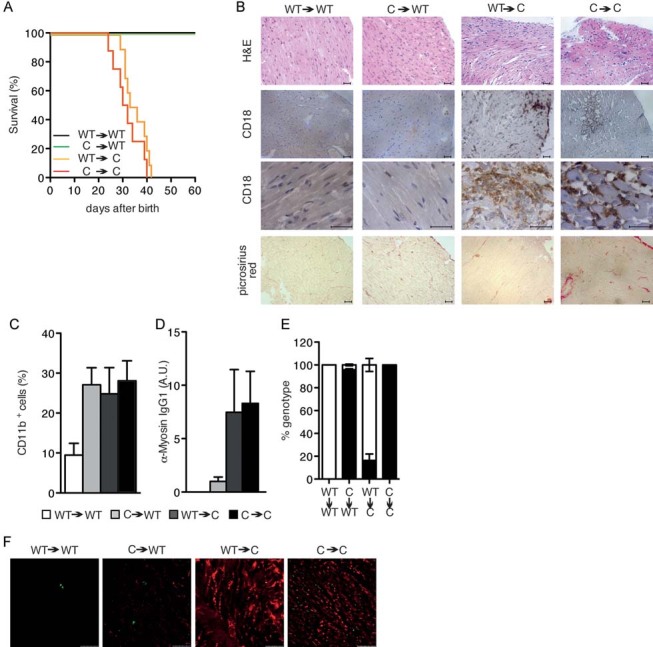
Reorganization of actin filaments results from clonal conversion. A. Relative increase in colony area of growing and terminal keratinocyte colonies after addition of EGF and in presence of Y27632 (10 µM). B. Relative increase in colony area of growing and terminal keratinocyte colonies after addition of EGF and in presence of (−)-blebbistatin (50 µM). C. pMLC immunostaining and rhodamine-phalloidin staining of cells localized at the periphery of a growing and a terminal colony incubated first for 1 h with Y27632 or (−)-blebbistatin before EGF (30 ng/ml) was added for 60 min. Scale bar: 2.5 µm. D. Distribution of angles of actin filaments against plasma membrane in cells localized at the periphery of growing and terminal colonies without EGF in presence of Y27632 or (−)-blebbistatin for 1 h. E,F. Relative increase in colony area in growing and terminal colonies after addition of EGF. Rapamycin was added for 1 hour (E) or 3 days (F) before EGF addition. G. pMLC immunostaining and rhodamine-phalloidin staining of localized at the periphery of growing and terminal colonies incubated with EGF for 60 min. Rapamycin was added for 1 h or 3 days before EGF addition. Scale bar: 20 µm. H. Distribution of angles of actin filaments against plasma membrane in cells localized at the periphery of growing and terminal colonies without EGF in presence of rapamycin for 1 h or 3 days.

To investigate the relation between the dynamics of a keratinocyte colony and clonal conversion, we isolated two progressively growing clones from a culture of normal human epidermal keratinocytes. These clones were then serially passaged in the presence of EGF. The rate of clonal conversion (switching from growing to terminal) increased with passages as expected; numerous progressive growing colonies were observed in early passages whereas numerous terminal colonies were observed in late passages. Results were identical in clonal and mass cultures, confirming that the switch in the response to EGF and the organization of actin filaments was linked to clonal conversion (Supporting Information Fig S5A and S5B). Next we investigated how colony dynamics is impacted by a ROCK inhibitor (Y27632), a molecule known to favour growing colonies (Chapman et al, 2010; McMullan et al, 2003; Terunuma et al, 2010). ROCK regulates both actin polymerization and MLC phosphorylation (Amano et al, 2010). Addition of Y27632 completely inhibited the response of both growing and terminal colonies to EGF (Fig 3A and Supporting Information Fig S6A). Interestingly, it drastically affected terminal colonies, which expanded similarly to growing colonies (Fig 3A and Supporting Information Fig S6A). Colonies treated with (−)-blebbistatin, a myosin ATPase inhibitor, also behaved similarly to those treated with Y27632 (Fig 3B). These results further confirmed that actomyosin interaction was essential for colony dynamics in response to EGF. Rhodamine-phalloidin staining of actin filaments revealed that the organization of the actin network in terminal colonies was now similar to that of a growing colony with Y27632, but not (−)-blebbistatin (Fig 3C and D). Y27632, but not (−)-blebbistatin, also diminished pMLC phosphorylation (Fig 3C and Supporting Information Fig S2C). However, Y27632 was not enough to rescue a paraclone from its commitment to terminal differentiation (Supporting Information Fig S6B). Together, these results indicated that inhibiting Rho signalling could remodel the actin filament network of a paraclone, but without consequence for its commitment to terminal differentiation. We further examined the effects of three small molecules, including an actin polymerization inhibitor (cytochalasin D), and two compounds that induce depolymerization of actin filaments (mycalolide B and bistheonelide A) on the growth capacity of human keratinocytes and clonal conversion. However, these molecules could not enhance clonal growth and colony-forming efficiency (CFE) significantly, as Y27632 did (Supporting Information Fig S7).

We also investigated how colony dynamics is impacted by rapamycin, another molecule that favours the formation of progressively growing colonies while decreasing the formation of paraclones (Brouard et al., in preparation). Addition of rapamycin, an inhibitor of mTORC1 signalling, for 1 h had no significant effect on expansion of growing colonies and shrinking of terminal colonies, regardless of EGF presence (Fig 3E), confirming that mTORC1 signalling had no impact on actin filament reorganization (Benjamin et al, 2011; Laplante & Sabatini, 2012). On the other hand, colonies treated with rapamycin for 3 days behaved similarly to those treated with Y27632 (Fig 3F). Indeed, long-term treatment of mammalian cells with rapamycin can suppress both mTORC1 and mTORC2 activities (Sarbassov et al, 2006; Supporting Information Fig S2E). Long-term treatment with rapamycin did not impact the organization of actin filaments, but significantly suppressed the EGF-induced MLC phosphorylation of myosin II (Fig 3G and H, and Supporting Information Fig S2E). This result further confirmed that EGF-induced MLC phosphorylation was essential for colony dynamics. These experiments first indicated that rapamycin had a different effect on colony behaviour when present for a short and a long time period; second, that long-term inhibition of mTOR signalling induced a response similar to the one observed when Rho signalling was inhibited.

### PI3K/Rac1 activity regulates organization of actin filaments and the dynamics of keratinocyte colonies

**Figure 4:**
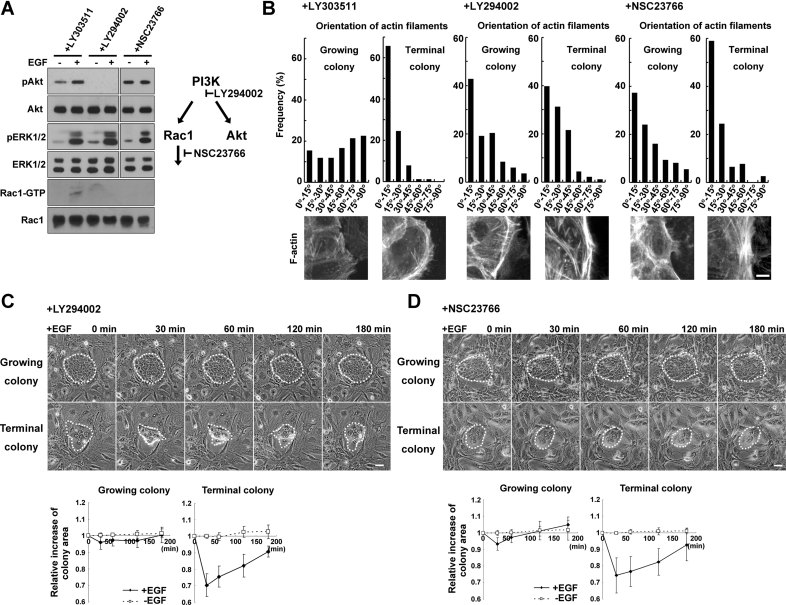
PI3K/Rac1 signalling regulates actin filament organization and the dynamic of human keratinocyte colonies in response to EGF. A. Human epidermal keratinocytes were cultured in presence of LY303511 (10 µM), LY294002 (10 µM) and NSC23766 (50 µM) for 1 h, proteins were extracted and Western blotting was performed to detect Akt, phosphorylated Akt (pAkt), ERK1/2, phosphorylated ERK (pERK1/2), and Rac1. Activated Rac1 was detected as a GTP-bound form (Rac1-GTP). B. Actin filaments were visualized by rhodamine-phalloidin staining of cells localized at the periphery of growing and terminal colonies cultured for 6 days without EGF. LY303511, LY294002, or NSC23766 was added for 1 h before cells fixed and stained. Upper panel shows distribution of angles of actin filaments against plasma membrane in cells localized at the periphery of growing and terminal colonies without EGF in presence of LY303511, LY294002 or NSC23766 for 1 h. Lower panel shows images of rhodamine-phalloidin staining of cells. Scale bar: 5 µm. C. Phase-contrast images (upper panel) and relative increase in colony area (lower panel) of growing and terminal keratinocyte colonies incubated for 1 h with LY294002 before EGF (30 ng/ml) was added. Colony edges are outlined with white dots. Scale bar: 50 µm. D. Phase-contrast images (upper panel) and relative increase in colony area (lower panel) of growing and terminal keratinocyte colonies incubated for 1 h with NSC23766 before EGF (30 ng/ml) was added. Scale bar: 50 µm.

Phosphatidylinositol 3-kinase (PI3K) signalling is involved in proliferation and differentiation of keratinocytes (Pankow et al, 2006; Sayama et al, 2002), as well as in actin cytoskeletal organization and cell motility in the HaCaT keratinocyte cell line (Pankow et al, 2006). A PI3K inhibitor (LY294002), but not its kinase inactive analogue (LY303511), inhibited PI3K-dependent Akt phosphorylation in cultured human keratinocytes (Fig 4A and Supporting Information Fig S8E). Without EGF, LY294002 induced the formation of stress fibres and cortical actin bundles parallel to the plasma membrane in both colony types (Fig 4B). Addition of LY294002 for 1 h, followed by EGF exposure, resulted in the shrinking of growing colonies, and in increased shrinking of terminal colonies (Fig 4C). Wortmannin, another PI3K inhibitor, acted similarly to LY294002 (Supporting Information Fig S8). PI3K controls a wide variety of downstream signalling pathways. Rac1, a member of Rho family of small guanosine triphosphatases (GTPases), is a downstream target of PI3K, and coordinates the dynamic organization of the actin cytoskeleton (Ridley et al, 1992). Phosphatidylinositol 3, 4, 5 phosphate (PIP3), a PI3K product, regulates a number of Rac-specific guanine nucleotide exchange factors (GEFs) that catalyse Rac activation (Cantrell, 2001). As expected, a Rac1 inhibitor (NSC23766) suppressed Rac1 activation in EGF exposed-cells, similarly to LY294002 (Fig 4A). Rac1 inhibition did not affect phosphorylation of ERK and Akt, the best-characterized downstream target of PI3K signalling (Fig 4A), but increased rhodamine-phalloidin staining of actin filaments at the cell periphery and induced actin bundles parallel to the plasma membrane in both growing and terminal colonies (Fig 4B), and resulted in the shrinking of growing colonies, and in increased shrinking of terminal colonies (Fig 4D). An Akt inhibitor (Akt inhibitor VIII) did not alter the organization of actin filaments and the response to EGF of both colony types (Supporting Information Fig S8). Collectively, these results indicated that PI3K/Rac1, but not Akt signalling, regulated actin filament organization and EGF-induced colony dynamics in human keratinocytes.

### Rac1 inhibition promotes clonal conversion

**Figure 5:**
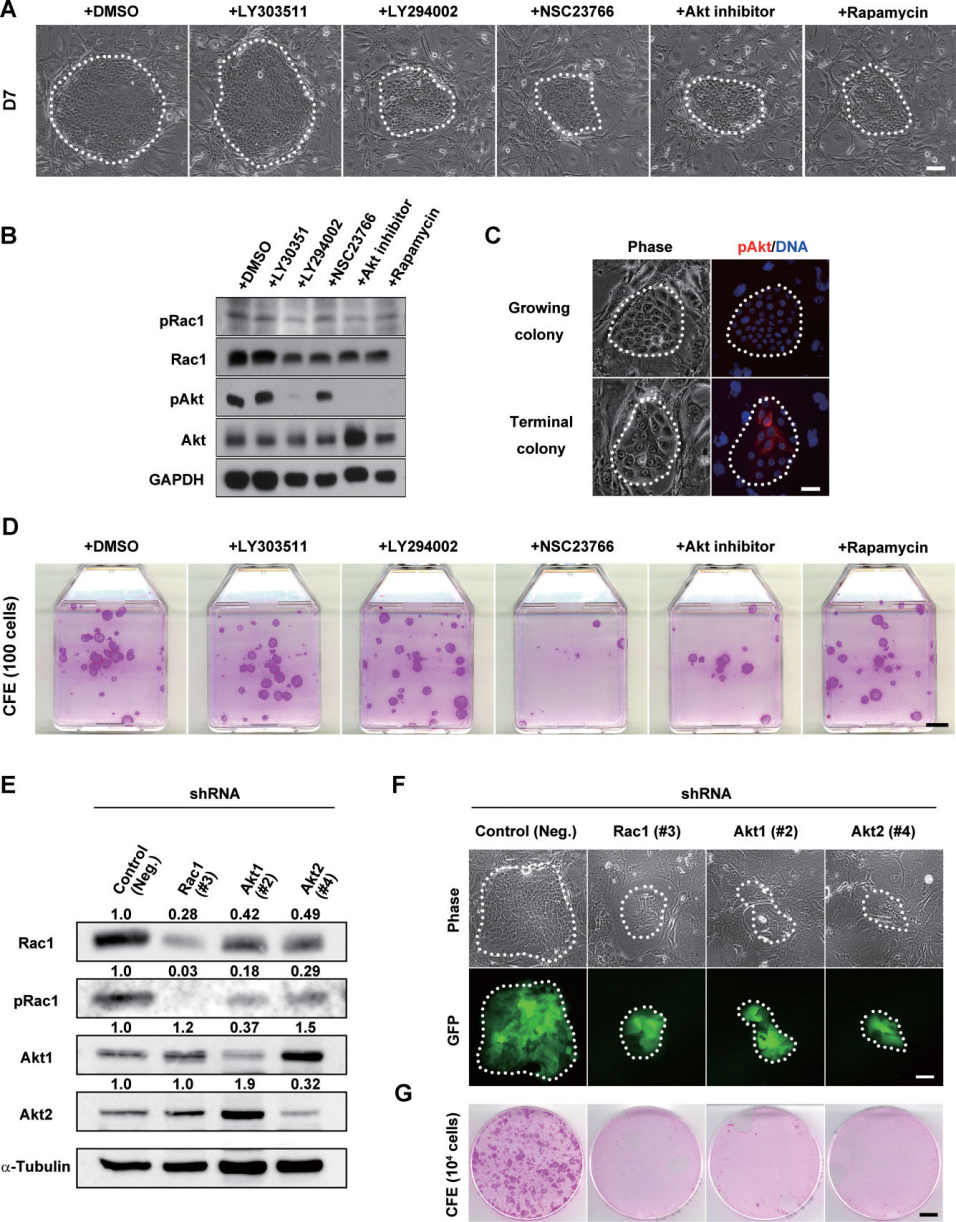
Rac1 activity inhibition promotes clonal conversion. A. Phase-contrast images of growing colonies of human keratinocytes. Signalling inhibitors LY303511 (10 µM), LY294002 (10 µM), NSC23766 (50 µM), Akt inhibitor (10 µM) and rapamycin (100 nM) were added after 4 days of cultivation; cells were cultured for 3 more days before the colonies were photographed. Colony edges are outlined with white dots. Scale bar: 100 µm. B. Western blotting of lysates from keratinocytes cultured in the conditions described in (A). C. Phase-contrast images and pAkt immunostaining of growing and terminal keratinocyte colonies. Scale bar: 50 µm. D. Determination of the colony-forming ability of keratinocytes cultured in presence of the different inhibitors for 3 days. Cultures were fixed and stained with rhodamine B after 11 days. CFE was performed as described in Material and Methods. Scale bar: 10 mm. E. Human epidermal keratinocytes were transduced with shRNA targeted to *Rac1*, *Akt1* or *Akt2* gene, proteins were extracted and Western blotting was performed to detect Rac1, pRac1, Akt1 and Akt2. Numerical values indicate relative density of bands normalized with density of α-tubulin bands. F. Upper panel shows phase-contrast images of colonies of human keratinocytes expressing Rac1, Akt1 or Akt2 shRNA. Lower panel shows expression of green fluorescence protein (GFP) confirmed the transcription of shRNA in presence of doxycyxline for 3 days. G. Determination of the colony-forming ability of shRNA-transduced keratinocytes cultured in presence of doxycycline for 3 days. 10^4^ cells were passaged and cultured without doxycycline before cultures were fixed and stained with rhodamine B after 9 days.

Inhibition of PI3K and Rac1 in holoclones and meroclones resulted in a switch in the organization of actin filaments and EGF-induced colony dynamics to a pattern similar to that of paraclones. Hence, we further investigated the role of PI3K signalling on clonal conversion in cultured human keratinocytes. Keratinocytes were seeded at clonal density (200 cells), and cultivated for 4 days before being exposed to EGF (10 ng/ml) and various signaling inhibitors (LY294002, NSC23766, Akt inhibitor and rapamycin). After a 3-day exposure, the growth capacity of keratinocytes was obviously impacted by the presence of the inhibitors, as the colonies were significantly smaller than in control cultures (Fig 5A). Colonies exposed to LY294002, Akt inhibitor and rapamycin mostly contained small cells, while NSC23766-treated colonies mostly contained large and flat cells. Interestingly, Akt inhibitor and rapamycin decreased the expression of INV, K1, K10, TG1 and DSG1, while NSC23766 increased INV expression (Supporting Information Fig S9A–C). After an 8-day exposure to various inhibitors, cultures were fixed and stained with rhodamine B. The number of keratinocyte colonies was not affected, but colony growth was impaired with inhibitors (Supporting Information Fig S9D). Rac1 and Akt are distinct downstream targets of PI3K signalling, however, Rac1 is also a substrate of Akt, and its phosphorylation by Akt decreases Rac1-GTP binding (Kwon et al, 2000). We confirmed that inhibition of PI3K and Akt significantly decreased Rac1 phosphorylation in cultured human keratinocytes (Fig 5B). Exposure to LY294002, Akt inhibitor and rapamycin was sufficient to inhibit Akt phosphorylation at Ser473, a target site of mTORC2 kinase activity (Sarbassov et al, 2005; Fig 5B). Importantly, phosphorylated Akt (Ser473) was only detectable in growth-arrested cells of terminal colonies, and not in proliferative small cells of growing colonies (Fig 5C). Similar results were also obtained with clones (Supporting Information Fig S5B). Collectively, these data suggested that Rac1 inhibition reduced the growth potential of cultured human keratinocytes. To further address this point, we cultured keratinocytes with different inhibitors for 3 days before cells were passaged at clonal density. The presence of a Rac1 inhibitor markedly reduced CFE from 25 ± 5.3% (mean ± sd) to 7.3 ± 1.3%, while increasing the number of terminal colonies (39 ± 2.6% to 70 ± 7.9%; Fig 5D). Keratinocytes grown in presence of an Akt inhibitor had a decreased CFE (7.8 ± 2.5%), but formed less terminal colonies (28 ± 4.7%) than cells grown without the inhibitor (Fig 5D). No significant changes were observed in cultures treated with a PI3K inhibitor or rapamycin (Fig 5D).

To further confirm these results, we next developed a doxycycline-inducible target-specific knockdown system using a single lentiviral vector (Supporting Information Fig S10A). Akt has three isoforms (Akt1/Akt2/Akt3) in mammalian cells, and Akt1 and Akt2 are predominantly expressed in human normal keratinocytes (Thrash et al, 2006). Therefore, we designed transgene cassettes for inducible expression of microRNA(miR)-typed short hairpin RNA (shRNA) targeted to *Rac1*, *Akt1* or *Akt2* gene, and screened and evaluated the knockdown efficiency of these shRNA constructs by Western blotting (Supporting Information Fig S10B–D and Fig 5E). As expected, Rac1 phosphorylation significantly decreased in the Rac1, Akt1 and Akt2 shRNA-expressing keratinocytes (Fig 5E). Surprisingly, knockdown of Akt1 and Akt2 also resulted in downregulation of Rac1 (Fig 5E). We next seeded keratinocytes at clonal density, and infected them with lentiviral vectors carrying the shRNA transgenes. Keratinocytes were further cultured for 4 days before shRNA expression was induced by treatment with doxycycline. In our system, the miR-typed shRNA sequence specific to a target gene is transcribed together with the gene of green fluorescent protein (GFP; Supporting Information Fig S10A), and shRNA-expressing cells are easily traced as GFP-positive cells. After a 3-day exposure to doxycycline, keratinocytes transduced with control shRNA gave rise to progressive growing colonies (Fig 5F). However, most of Rac1, Akt1 and Akt2 shRNA-transduced colonies showed terminal phenotype (Fig 5F). Subsequently, shRNA-expressing keratinocytes were passaged without doxycycline. The temporal knockdown of Rac1, Akt1 and Akt2 markedly reduced CFE while increasing the number of terminal colonies, and the cultures could not be maintained any more (Fig 5G). Collectively, these results indicated that Rac1 was necessary for maintaining the growing phenotype, and that Akt kinase activity was necessary to promote the terminal phenotype, while Akt expression was required for Rac1 expression.

## DISCUSSION

**Figure 6:**
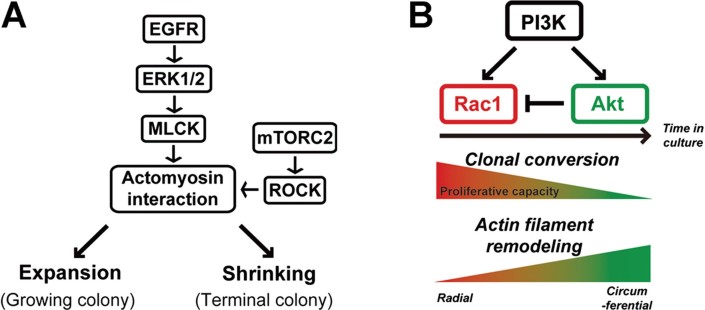
EGFR/ERK/MLCK and PI3K/Rac1/Akt signalling. A. A schematic representation of EGFR/ERK/MLCK signalling on the dynamics of human keratinocyte colonies. B. A model on how PI3K/Rac1/Akt signalling can impact clonal conversion. A decrease in Rac1 through Akt signalling promotes clonal conversion and remodelling of actin filaments.

A colony initiated by a human keratinocyte stem cell grows by a combination of cell migration and multiplication, and requires EGFR ligands (Barrandon & Green, 1987b; Coffey et al, 1987; Rheinwald & Green, 1977). Activation of EGFR signalling immediately induces the lateral expansion of colonies that are actively multiplying (Barrandon & Green, 1987b), and also enhances the helical migration of individual keratinocytes seeded onto a fibrin matrix (Ronfard & Barrandon, 2001). Moreover, EGFR signalling is important for proliferation of keratinocyte stem cells through Lrig1 (Jensen & Watt, 2006; Jensen et al, 2009), for the re-epithelialization of an epidermal wound (Barrientos et al, 2008; Higashiyama & Nanba, 2005; Gurtner et al, 2008; Pastore et al, 2008), and for the massive *ex vivo* expansion of human keratinocytes for regenerative medicine (De Luca et al, 2006; Rochat et al, 2012). Our results now demonstrate that the differing organization of the actin filament network in colonies formed by human keratinocyte stem cells (holoclones) and TA cells (paraclones) set up the stage of their response to EGF through EGFR/ERK/MLCK signalling (Fig 6A).

The radial distribution of actin filaments preserved by Rac1 activity in holoclones is required for the expansion of progressively growing colonies in response to EGF. This EGF-induced colony dynamics results from centrifugal migration of the cells located at the periphery of growing colonies, and is essential for sustained growth of keratinocyte colonies (Barrandon & Green, 1987b). We also demonstrate here that inhibition of EGFR/ERK signalling-induced colony expansion induces the expression of terminal differentiation markers. Furthermore, EGF increased the clonogenicity, but not multiplication, of human epidermal keratinocytes forming small colonies, which dynamics we examined in this study (Rheinwald & Green, 1977). These data suggest that EGF-induced colony dynamics defined by actin filament organization is involved in clonal growth and conversion. This study shows that remodelling of actin filaments of paraclones to a holoclones-type pattern by Y27632 cannot rescue a paraclone from its commitment to terminal phenotype, and that inhibition of actin polymerization and depolymerization of actin filaments are not sufficient to reduce, stop and/or reverse clonal conversion. However, a switch in the organization of actin filaments from holoclone- to paraclone-type by Rac1 inhibition decreases clonal growth, and promotes clonal conversion. These experiments explain that radial distribution of actin filaments is essential for holoclone-colony dynamics and maintenance of growth capacity of holoclones, but not sufficient for restoration of growth capacity of paraclones.

Continuous inhibition of Rho signalling (Chapman et al, 2010; McMullan et al, 2003; Terunuma et al, 2010), and continuous inhibition of mTOR signalling by rapamycin (Brouard et al., in preparation) favour the formation of progressively growing colonies, while decreasing the formation of paraclones. mTOR signalling plays an important role in balancing cell behaviour (Benjamin et al, 2011; Ma & Blenis, 2009; Laplante & Sabatini, 2012) through mTORC1, which is rapamycin sensitive and acts on translation, and mTORC2, which is rapamycin insensitive and regulates actin filament organization (Jacinto et al, 2004) and actomyosin contractility through Rho/ROCK signalling (Liu et al, 2010). However, little is known on how mTOR signalling impacts human keratinocyte stem cell behaviour (Javier et al, 1997). Our results demonstrate that continuous exposure of a keratinocyte culture to rapamycin inhibits phosphorylation of the MLC and impacts colony dynamics. This indicates that rapamycin can also impact mTORC2, as it has been recently demonstrated (Hosking, 2012; Lamming et al, 2012). Moreover, our results demonstrate that the response of keratinocyte colonies to continuous inhibition of ROCK signalling is strikingly similar to that of colonies exposed to rapamycin, thus suggesting the existence of a functional link between Rho/ROCK and mTORC2 signalling and the significance of phosphorylation of MLC for keratinocyte stem cell maintenance.

PI3K signalling impacts cell motility and actin filament organization, as well as proliferation and differentiation in human keratinocytes (Pankow et al, 2006; Sayama et al, 2002). Moreover, activation of Akt induces keratinocyte terminal differentiation (Calautti et al, 2005; Janes et al, 2009; Thrash et al, 2006), promotes growth arrest (Calautti et al, 2005), and results in clonal conversion (Janes et al, 2009). In contrast, Rac1 is required for clonal growth of keratinocytes (Benitah et al, 2005), and a decreased expression of Rac1 induces keratinocyte differentiation (Nikolova et al, 2008). Our findings indicate that activation of Akt signalling becomes predominant during serial cultivation, with the consequence of decreasing Rac1 activity. This results in remodelling of actin filaments, and in clonal conversion. Hence, we suggest the existence of a molecular circuitry wiring Rac1 to PI3K and Akt (Fig 6B), the modulation of which impacts stem cell behaviour by either enhancing or decreasing clonal conversion. This model presents that PI3K is required for both Rac1 and Akt activity and that inhibition of PI3K causes both Rac1 inactivation and activation, suggesting that the level of Rac1 activity is stable even when a PI3K inhibitor is present and/or that PI3K inhibition does not affect a balance between Rac1 and Akt activity. This can be explained our data why PI3K inhibition does not decrease CFE. Surprisingly, shRNA knockdown of Akt1 and Akt2 results in the decreased expression of Rac1 protein, and accelerates clonal conversion. An Akt inhibitor decreases colony-forming ability of keratinocytes, but not Rac1 expression. This result suggests the existence of kinase activity-independent regulation of Rac1 expression by Akt1/2 in keratinocytes. It is likely that Akt1/2 is required for Rac1 signalling and that shRNA knockdown of Akt1/2 interferes with Rac1 signalling and decreases Rac1 protein levels consequently. This point should be considered for regulating keratinocytes stem cells.

In conclusion, our data demonstrate that remodelling of actin filaments and inhibition of actomyosin contractility can impact stem cell behaviour through the modulation of clonal conversion. This is of paramount importance for regenerative medicine.

## MATERIALS AND METHODS

### Cell culture

Human keratinocyte YF29 cells and normal human epidermal keratinocytes (KURABO) were from foreskin of newborns. Frozen cells were thawed and cultivated at clonal density on a feeder layer of irradiated 3T3-J2 cells at 37°C as described (Rheinwald & Green, 1975; Rochat et al, 1994). The medium was changed every 4 days. Cells were used between passage 4 and 10. For determination of CFE, 100 keratinocytes were cultured as previously described. Cultures were fixed in 3.7% buffered formaldehyde and stained with 1% rhodamine B, and keratinocyte colonies were counted under a binocular microscope.

### Inhibitors and antibodies

Pharmacological inhibitors and Primary antibodies used for immunofluorescence and Western blotting were described in Supporting Information Table S1 and S2, respectively.

### Measurement of keratinocyte colony area and time-lapse imaging

Human epidermal keratinocytes were seeded at clonal density in a 35 mm size cell culture dish (Corning) with irradiated 3T3-J2 cells, and grown for 6 days without EGF. Cells were then cultivated with or without EGF (30 ng/ml) for 3 h. Small molecule inhibitors were added 1 h before starting the EGF treatment. Microscopic colonies were photographed at various times after EGF stimulation and their area was then measured with MetaMorpho (Molecular Devices) or Volocity (PerkinElmer) using a graphic tablet (Wacom). Mean size (±SD) was calculated on measurements obtained from at least five colonies. For time-lapse imaging, cultures were maintained at 37°C and 10% CO_2_ in a chamber mounted on an Axiovert 200 M microscope (Zeiss). Images were obtained at 5 min intervals for 180 min.

### Measurement of actin filament orientation

An angle of single actin filament against the plasma membrane in a cell localized at the periphery of a keratinocyte colony was defined as an acute crossed-axes angle (0° ≤ *θ* ≤ 90°) between a line linking cell–cell contact points at colony edge, and an angle of single actin filament in a cell. See also Supporting Information Fig S4. The angles of three well-defined actin filaments in individual keratinocytes were measured with Volocity (PerkinElmer) using a graphic tablet (Wacom). The distribution was obtained from at least 75 well-defined actin filaments in more than 25 cells of growing or terminal colonies in each experimental condition.

### Immunofluorescence microscopy

YF29 cells were seeded at clonal density in a 35 mm cell culture dish with irradiated 3T3-J2 cells, and grown for 6 days without EGF. In some cases, the cells were stimulated with 30 ng/ml EGF for various times in each experiment. After that, the cells were washed twice with cold phosphate-buffered saline (PBS), and fixed in 4% paraformaldehyde in PBS at 4°C for 10 min. The cells were treated with 0.5% Triton X-100 in PBS for 10 min and subsequently with 1% BSA. The cells were then incubated with primary antibodies for 1 h at room temperature (RT) or overnight at 4°C, washed in PBS, and incubated with Alexa Flour 488-conjugated goat anti-mouse IgG or Alexa Flour 568-conjugated goat anti-rabbit IgG (Dako) for 1 h at RT. In some cases, the cells were treated with rhodamine-phalloidin (Invitrogen) for 30 min at RT. After a wash with PBS, the cells were incubated with Hoechst 33258 (Molecular probes), mounted with a fluorescent mounting medium (Dako), and examined with a Carl Zeiss Axioplan 2 epifluorescence microscopy.

### Western blotting

YF29 cells were seeded in a 60 mm cell culture dish (Falcon) with irradiated 3T3-J2 cells, and grown for 6 days without EGF. In some cases, the cells were stimulated with 30 ng/ml EGF for various times in each experiment. After that, the cells were washed twice with cold-PBS, and lysed with 1% Triton-X 100, 5 mM EDTA in PBS containing protease inhibitor cocktail (Roche) and phosphatase inhibitor cocktail 1 (Sigma). For the detection of MLC and phosphorylated MLC, keratinocytes were lysed with SDS sample buffer. Equal amounts of proteins were dissolved in SDS sample buffer and separated by SDS–PAGE. Proteins in the gels were transferred to nitrocellulose membranes (Schleicher & Schuell). After the blocking with 5% skimmilk or 5% BSA in PBS, the membranes were immunoblotted against primary antibodies overnight at 4°C. After a wash with 0.05% Tween-20 in PBS, the membranes were incubated with horseradish peroxidase (HRP)-conjugated anti-rabbit or anti-mouse antibody (Jackson Immunoresearch) for 1 h at RT. The membranes were washed with 0.05% Tween-20 in PBS, treated with an enhanced chemiluminescence substrate for detection of HRP (Pierce) for 5 min, and exposed to Kodak BioMAX MR film.

### Quantitative RT-PCR

Total RNA was extracted from cultured keratinocytes with TRIzol reagent (Invitrogen). cDNAs were synthesized from 1 µg of total RNA with SuperScript III (Invitrogen) and hexamer random primers (Invitrogen), according to manufacturers' instructions. cDNAs were adjusted to equal levels by PCR amplification with primers for TBP1. Primers were designed to ensure the uniqueness for each gene, using LightCycler Probe Design 1.0 software and NCBI BLAST. The list of primers used is described in Supporting Information Table S3. Quantitative PCR was performed using LightCycler FastStart DNA Master SYBR Green I reagents, and a LightCycler System piloted by LightCycler 3.5 software (Roche Diagnostics). Differences between experimental samples and controls were calculated on the basis of the 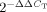 method.

**The paper explained**

**PROBLEM**

Human epidermal keratinocyte stem cells are critical for regenerative medicine for burns and genetic disorders. In serial culture, the keratinocyte stem cells progressively lose their proliferative capacity and become cells with limited growth (transient amplifying cells), a phenomenon termed clonal conversion that negatively impacts the culture lifespan and the success of cell transplantation. Although, clonal conversion is dramatically increased by stress, an inadequate microenvironment (niche), serial cultivation and age of donor, little is known on the underlying molecular mechanisms.

**RESULTS**

Here we show that colonies of keratinocyte stems cells differ from those of transient amplifying cells in their organization of actin filament organization, and in their response to epidermal growth factor (EGF). Moreover, inhibition of PI3K or Rac1 in keratinocyte stem cells induces the reorganization of actin filaments and an EGF response that are similar to those of transient amplifying cells. Importantly, continuous Rac1 inhibition in keratinocyte stem cells results in clonal conversion and reduction of growth potential. Together, out data demonstrate that human epidermal keratinocyte stem cells are maintained in culture by EGF signalling and actin filament dynamics in a cooperative manner and we propose the existence of a molecular circuitry that governs clonal conversion.

**IMPACT**

Modulation of signalling pathways that impact clonal conversion will improve stem cell self-renewal and engraftment, together with the long-term maintenance of the regenerated epidermis in transplanted patients.

### Rac1 activity assay

GTP-bound Rac1 was detected by using Rac1 activation assay kit (Millipore), according to manufacturer's instructions.

### Plasmid DNA construction for lentiviral expression vector

The HIV-based self-inactivating lentiviral expression vector plasmid CS-CA-MCS (Miyoshi et al, 1998) was provided by RIKEN BioResource Center, Japan. Three plasmids, pTet-On-Advanced vector, pTRE-Tight vector (Clontech laboratories), and pcDNA 6.2-GW/EmGFP-miR vector (Invitorgen) were obtained, and their components were utilized to construct a transgene cassette consisting of two expression units for inducible gene knockdown in this study. The cDNA fragment of reverse Tet-controlled transactivator (rtTA) was recovered from the pTet-On-Advanced vector and ligated into the pIRES vector (Clontech), upstream the sequence of internal ribosome entry site (IRES). The blasticidin-resistance gene (Bsd) was ligated into this plasmid, downstream the IRES sequence, resulting in the generation of prtTA-IRES-Bsd, containing one transgene unit. On the other hand, a *Dra* I-*Pv*u II fragment of the pcDNA 6.2-GW/EmGFP-miR vector containing EmGFP cDNA and pre-miRNA sequence was recovered and inserted into the pTRE-Tight vector, downstream the Tet-responsive promoter. This resulted in the vector pTRE-EmGFP-miR, which contained another transgene unit. Finally, the fragment of the rtTA-IRES-Bsd was inserted into the lentivirus expression vector plasmid, CS-CA-MCS, directly downstream of the CAG promoter sequence, and then another unit, the TRE-EmGFP-miR fragment, was inserted into the resulting plasmid, between the Bsd and WPRE sequence. The TRE-EmGFP-miR unit was arranged in the opposite direction as the CAG-driven rtTA-IRES-Bsd unit, yielding a new single lentivirus expression plasmid for inducible gene silencing, CS-CA-rtTA-IRBsd-TRE-EmGFP-miR (Supporting Information Fig S10A).

### Lentiviral vector for doxycycline-dependent inducible gene knockdown

In order to construct the lentivial vectors for doxycycline-dependent inducible silencing of the human *Rac1*, *Akt1* and *Akt2* gene expression, a set of synthetic oligonucleotides in each gene designed by BLOCK-iT miR RNAi technologies of Life Technologies, Inc., was obtained, respectively. The 21nt target sequences were described in Supporting Information Table S4. The double-strand DNA was inserted between the 5-miR and the 3′-miR flanking sequences of the vector plasmid. The miR-negative control sequence presented by Life Technologies, Inc., was used to construct a negative control vector. Using these plasmid DNAs, lentiviral particles were prepared by standard transfection procedures with packaging plasmid DNAs (pCAG-HIVgp and pCMV-VSV-G-RSV-Rev; RIKEN BioResource Center), and used to introduce the transgene into 1 × 10^5^ cells. After transduction of the transgenes at a multiplicity of infection of 5, the cells were treated with 100 nM doxycyline to induce silencing of the gene, and subsequently analysed.

## Author contributions

DN, FT and YB designed the experiments; DN and FT performed most of experiments and analysed the data; NM and SM developed the inducible knockdown lentivirus system and produced lentiviruses; YB and SH interpreted the data with DN and supervised the project; All authors discussed the results and commented on the manuscript; DN and YB wrote the manuscript.

## Notes

As a service to our authors and readers, this journal provides supporting information supplied by the authors. Such materials are peer reviewed and may be re-organized for online delivery, but are not copy-edited or typeset. Technical support issues arising from supporting information (other than missing files) should be addressed to the authors.
