# Supplementary material for: Actin filament dynamics impacts keratinocyte stem cell maintenance
Source: EMBO Mol Med. 2013 Apr 2;5(4):640–53. doi: 10.1002/emmm.201201839 (PMC3628097; doi:10.1002/emmm.201201839)
Supplement: Supplementary file 1 [file emmm0005-0640-sd1.pdf]

EMBO Molecular Medicine

Actin Filament Dynamics Impacts Keratinocyte Stem Cell Maintainance

Daisuke Nanba, Fujio Toki, Natsuki Matsushita, Sachi Matsushita, Shigeki Higashiyama, and Yann Barrandon

Corresponding author: Daisuke Nanba, Ehime University

Review timeline:

Submission date:	07 August 2012
Editorial Decision:	19 September 2012
Revision received:	18 December 2012
Editorial Decision:	27 January 2013
Revision received:	13 February 2013
Accepted:	14 February 2013

Transaction Report:

(Note: With the exception of the correction of typographical or spelling errors that could be a source of ambiguity, letters and reports are not edited. The original formatting of letters and referee reports may not be reflected in this compilation.)

Editor: Anneke Funk / Roberto Buccione

1st Editorial Decision 19 September 2012

Thank you for the submission of your manuscript "Actin Filament Dynamics Impacts Keratinocyte Stem Cell Maintainance" to EMBO Molecular Medicine and please accept my apologies for the delayed reply. We have now finally received the reports from the three referees whom we asked to review your manuscript. You will see that they find the topic of your manuscript potentially interesting. However, they also raise significant concerns on the study, which should be addressed in a major revision of the manuscript.

Importantly, both Reviewer #2 and #3 highlight that RNAi should be used to substantiate the conclusions derived from inhibitor studies. The Reviewers also note that, in addition to a number of technical concerns, the readability of the manuscript for a broad audience should be improved.

Given the balance of these evaluations, we feel that we can consider a revision of your manuscript if you can convincingly address the issues that have been raised within the time constraints outlined below. However, we also realize that addressing all of the referees' criticisms might require a lot of additional time and effort and be technically challenging. I am uncertain whether you will be able (or willing) to return a revised manuscript within the 3 months deadline and I would also understand your decision if you chose to rather seek rapid publication elsewhere at this stage. Should you decide to embark on such a revision, revised manuscripts should be submitted within three months of a request for revision. They will otherwise be treated as new submissions unless arranged otherwise with the editor.

***** Reviewer's comments *****

Referee #1:

Cultured human epidermal stem are already used in regenerative medicine including burns and genetic disorders. Understanding the mechanisms of clonal conversion of stem cells towards transient amplifying cells will help improve their long-term engraftment in these patients.

In this article, Nanba et al assessed the impact of actin filament dynamics and contraction on the maintenance of keratinocyte stem cells and clonal conversion in vitro. Very interstingly, they showed that holoclones and paraclones have different responses to EGF stimulation; holoclones show progressive growing while paraclones shrink. MEK1/2 inhibitors or Myosin light chain kinase inhibitor could block this response to EGF. They then show that actin filaments were differently distributed in the peripheral cells of holoclones and paraclones. They also observed that the response to EGF was linked to clonal conversion. Interestingly inhibiting Rho signaling inhibited the response to EGF and stimulated the growth terminal colonies but without consequence toward their commitment to terminal differentiation. Long-term mTOR inhibitor rapamycin had the same effect on colony growth. Finally, they cultured human keratinocytes in the presence of Rac1, PI3K or Akt inhibitor and conclude that Rac1 maintains colony growth while Akt promote the formation of paraclones.

This is an interesting study with clear medical relevance. The methodology is appropriate and the data shown convincing. Altogether, I recommend publication of this manuscript in EMBO molecular medicine pending the authors address the following comments.

Specific comments

- Figure 1, 2 and 3: The effect of EGF on the growth of holoclones and shrinking of paraclones is observed over 3 hours. The effect of MEK inhibitors, ROCK inhibitor, rapamycin and the inhibitors too. Based on these short-term observations, the authors built their working hypothesis and conclusions on the remodeling and contraction of actin filaments.
However, clonal growth and conversion occurs over long-term culture (over several passages and several days) and are governed by the balance between proliferation and differentiation. This can be seen in figure 5A where the size of the colonies is obviously dependent on the number of cells within the colony and not the contraction of the cells.

The term "growth" is then inappropriate to use for holoclones and should be replace by "dilation" (as opposed to "shrinking" of paraclones)

The authors correlate the actin filament orientation with the growing capacities of the colonies. Can the author use different inhibitor to convert paraclones into holoclones and correlate this growing capacity with the orientiation of actin filaments within the colonies?

- Figure 3: The inhibition of Rho signaling and long-term treatment with rapamycin inhibited holoclone growth/dilation and paraclone shrinking. Nevertheless, inhibition of Rho signaling remodeled actin filament network of paraclones without consequence for their commitment to terminal differentiation, while long-term treatment with rapamycin did not impact actin filament organization. Both inhibited phosophorylation of Myosin light chain.

This raised the question about whether the actin filament organization and their contraction are not key players in clonal growth and clonal conversion at least in this set of experiments. The authors should provide a clear explanation for these phenomena.

- Figure 5: The effect of Akt and Rac1 inhibition should be tested in long term treatment and assess the rate of clonal conversion.

Minor comments

Higher magnification of the IF showing the actin organization should be provided.

The manuscript is sometime difficult to read and would probably benefit from being checked and edited by a non-specialist to ensure readability and clarity for a broad audience.

Referee #2 (Comments on Novelty/Model System):

suggestions for improvement are listed in the main comments to authors

Referee #2 (General Remarks):

The manuscript by Namba and co-workers analyse the actin cytoskeleton organisation of primary keratinocytes. They propose that holo clones and paraclones can be differentiated by specific aspects of the actin filament structures, which leads to changes in colony behaviour, response to EGF and proliferative capacity of different clones. The manuscript is well written and figures of high quality. However, this reviewer has not been persuaded by the argument or conclusions in the manuscript. The phenotypes observed can have alternative explanations that do not involve clone conversion. The data are thus over-interpreted and not supported by the results shown.

The actin cytoskeleton is very dynamic and can remodel very quickly in response to different stimuli.
Importantly, actin organisation in keratinocytes is very variable, and thus without a population study on percentage of cells that show a particular organisation of actin, the data is not convincing. I understand that time lapse experiments are laborious, but the quantification and classification of cells that have a particular organization of the cytoskeleton can be done in fixed samples.

Delineation of pathways is achieved by long term treatment with inhibitors that per se can clearly have toxic, non-specific effects in cells. First, these inhibitors are not specific to the proteins mentioned, and thus contribution of other proteins for the phenotype cannot be excluded (see papers by Bain et al 2003 and 2007). Confirmation of the results with RNAi is then very important to substantiate the conclusions. Second, if looked at face value, the same phenotypes on cytoskeletal organization shown for inhibition of Akt, PI3K, etc in different cell types are now shown again in keratinocytes and proposed to regulate clone conversion. It could be that enlargement of colony area just reflects that (i) spreading of the keratinocytes in general, and (ii) the perturbed response of terminal colonies is because of a rewiring of signalling related to Rac signalling to spreading (rather than clone conversion).

Analysis of MLC phosphorylation following treatment with different inhibitors is simplistic and contains flaws. For example some of the inhibitors used disassemble actin bundles and thus obviously there will be no phosphorylated MLC staining on bundles because the bundles are not there. This conclusion can only be made if pMLC is reduced without perturbation of the actin fibers.

Figure 2a- cells shown are not comparable. On top, the cell lies in the middle of the colony, surrounded by neighbouring cells, while in the bottom panel, cell is at the periphery of the colony, and thus able to protrude lamella.

Image used for control +EGF in figure 3C is the same used previously in Fig.2D.

Authors should specify if de-lipidated BSA was used to add to EGF onto cells. If not, standard BSA contains lipids that can stimulate Rho and MLC phosphorylation.

Referee #3 (Comments on Novelty/Model System):

This paper would be greatly improved by clearer explanation of the experiments, the rationale for which is hard to follow without a good knowledge of the signalling pathways involved. While the actin results are of some interest, the observation of the role of Rac1 in human epidermal stem cells is the most important point. This may not be directly linked with the actin effect.

© EMBO

Referee #3 (General Remarks):

Primary cultures of human keratinocytes are important in regenerative medicine as this system is in routine use for ex vivo expansion for autologous transplantation in patients with burns. This manuscript examines the relationships between the actin cytoskeleton and clonogenic potential in primary cultures of human keratinocytes, in order to better define the events associated with a loss of stem cell self renewal that limits the expansion of cells in culture. The investigations on actin cytoskeleton in different colony types in this study lead to the identification of Rac1 as essential for sustaining the growth of stem cell derived colonies, a finding which extends previous observations in mice to the human system.

Abstract:

It would be worth explaining the role of cultured keratinocytes in therapy and the terms holoclone and paraclone more clearly as these would not be familiar to a non specialist reader. The link between EGF and RAC signaling could also be explained.

Results:

The authors begin by demonstrating that terminally differentiating colonies shrink in response to EGF treatment whilst larger proliferating colonies expand. The expansion of larger colonies is blocked by MEK and MLCK inhibition but the shrinking of smaller colonies is refractory to these agents. It would be helpful to have a line or two explaining the EGFR/ERK/MLCK pathway and the rationale for using the inhibitors for readers not familiar with this aspect of signaling.

Next the actin cytoskeleton is examined (Fig. 2) and differences in F actin organization observed between growing and differentiated colonies in the presence of EGF and/or inhibitors. The relationship between actin organization and colony type is shown to be independent of passage number (Fig S2).

In Figure 3 the effect of drugs that increase keratinocyte clonogenicity is examined. The ROCK inhibitor Y27632 is found to block the effects of EGF in both colony types. Short term treatment with rapamycin has little effect, whereas long term treatment, argued to act on mTORC2 blocks the effect of EGF on both colony types. The activity of these agents should be confirmed by Western blotting, as has been done for the other inhibitors, particularly to demonstrate the difference between short vs long term rapamycin treatment.

In Figure 4 PI3K signalling is investigated, again using inhibitors. PI3K or Rac1 inhibition reorganises actin in both colony types, blocks the EGF response of growing colonies and augments the EGF effect on terminally differentiating colonies.

Figure 5 demonstrates that Rac1 inhibition potently inhibits colony growth, triggering the transition from actively growing to terminally differentiating colonies. Akt inhibition has a similar effect. Given that the important conclusion of this study is the link between Rac1 and clonogenicity, confirming the role of Rac1 with shRNA knockdown would strengthen the argument.

In general the explanation as to why each experiment is performed could be greatly improved for readers unfamiliar with the pathways involved.

The study concludes "remodeling of actin filaments and inhibition of actomyosin contractility can impact stem cell behavior through the modulation of clonal conversion". Whilst actin remodelling is associated with differentiation it is not clear that effects on the actin cytoskeleton itself underlie conversion.

1st Revision - authors' response 18 December 2012

For Referee #1

Cultured human epidermal stem are already used in regenerative medicine including burns and genetic disorders. Understanding the mechanisms of clonal conversion of stem cells towards transient amplifying cells will help improve their long-term engraftment in these patients.

In this article, Nanba et al assessed the impact of actin filament dynamics and contraction on the maintenance of keratinocyte stem cells and clonal conversion in vitro. Very interestingly, they showed that holoclones and paraclones have different responses to EGF stimulation; holoclones show progressive growing while paraclones shrink. MEK1/2 inhibitors or Myosin light chain kinase inhibitor could block this response to EGF. They then show that actin filaments were differently distributed in the peripheral cells of holoclones and paraclones. They also observed that the response to EGF was linked to clonal conversion. Interestingly inhibiting Rho signalling inhibited the response to EGF and stimulated the growth terminal colonies but without consequence toward their commitment to terminal differentiation. Long-term mTOR inhibitor rapamycin had the same effect on colony growth. Finally, they cultured human keratinocytes in the presence of Rac1, PI3K or Akt inhibitor and conclude that Rac1 maintains colony growth while Akt promote the formation of paraclones.

This is an interesting study with clear medical relevance. The methodology is appropriate and the data shown convincing. Altogether, I recommend publication of this manuscript in EMBO molecular medicine pending the authors address the following comments.

Specific comments

- Figure 1, 2 and 3: The effect of EGF on the growth of holoclones and shrinking of paraclones is observed over 3 hours. The effect of MEK inhibitors, ROCK inhibitor, rapamycin and the inhibitors too. Based on these short-term observations, the authors built their working hypothesis and conclusions on the remodelling and contraction of actin filaments.
However, clonal growth and conversion occurs over long-term culture (over several passages and several days) and are governed by the balance between proliferation and differentiation. This can be seen in figure 5A where the size of the colonies is obviously dependent on the number of cells within the colony and not the contraction of the cells.

A: Keratinocytes start to migrate centrifugally within 15 min after EGF addition, resulting in the expansion of progressively growing colonies. This EGF-induced cell migration is essential for sustained growth of keratinocyte colonies, as we demonstrated previously (Barrandon and Green, Cell 50, 1131-1137, 1987). In this study, we also indicated that EGFR/ERK/MLCK signaling is essential for colony expansion that results from centrifugal cell migration of growing colonies, and that keratinocytes started to differentiate when this colony expansion was suppressed by ERK inhibition (new Fig S3). Furthermore, EGF increased the clonogenicity, but not multiplication, of human epidermal keratinocytes forming small colonies, which dynamics we examined in this study (Rheinwald and Green, Nature 265, 421-424, 1977). The effects on the successful expansion of human keratinocytes show that EGF cannot be viewed as a simple mitogen, as has generally been supposed. These data strongly suggest that EGF-induced colony dynamics (short-term behaviors) governs clonal growth and conversion, which are controlled by a balance between proliferation, differentiation, and senescence (long-term behaviors). We have discussed this point in “Discussion” of the text.

The term "growth" is then inappropriate to use for holoclones and should be replace by "dilation" (as opposed to "shrinking" of paraclones)

A: In this manuscript, we use the term “expansion”, but not “growth”, for EGF-induced holoclone colony dynamics (“shrinking” for paraclones). We previously used the term “expansion” for increase in colony area after EGF addition (Barrandon and Green, Cell 50, 1131-1137, 1987). In consideration of consistency with our previous paper, we have used the term “expansion”, but not “dilation”, in this revised manuscript.

We used the term "growth" for a long-term phenotype of keratinocyte colonies. However, this term is not used for short-term colony dynamics. According to you suggestion, we have made sure that we appropriately used the terms "expansion" and "growth" in this revised manuscript.

The authors correlate the actin filament orientation with the growing capacities of the colonies. Can the author use different inhibitor to convert paraclones into holoclones and correlate this growing capacity with the orientation of actin filaments within the colonies?

A: We have already selected three small molecules, including an actin polymerization inhibitor (cytochalasin D), and two compounds that induce depolymerisation of actin filaments (mycalolide B and bistheonelide A). We examined the effects of different concentrations of these molecules on the growth capacity of human keratinocytes and clonal conversion. However, these molecules could not convert paraclones into holoclones. Remodelling of actin filaments from paraclone- to holoclone-type was not sufficient to reduce, stop, and/or reverse clonal conversion, as also shown in experiments with Y27632 (new Fig 3C and 3D and new Fig S6B). We have discussed this point in the "Discussion" section.

- Figure 3: The inhibition of Rho signalling and long-term treatment with rapamycin inhibited holoclone growth/dilation and paraclone shrinking. Nevertheless, inhibition of Rho signalling remodelled actin filament network of paraclones without consequence for their commitment to terminal differentiation, while long-term treatment with rapamycin did not impact actin filament organization. Both inhibited phosphorylation of Myosin light chain.

This raised the question about whether the actin filament organization and their contraction are not key players in clonal growth and clonal conversion at least in this set of experiments. The authors should provide a clear explanation for these phenomena.

A: As you mentioned, Y27632 could not rescue a paraclone from its commitment to terminal phenotype, even if this inhibitor remodelled actin filament organization of paraclones to that of holoclones (new Fig 3D and Supporting Information Fig S6B). This means that remodelling of actin filaments from paraclone- to holoclone-type is not involved in decreased clonal conversion. However, remodelling of actin filaments from holoclone- to paraclone-type by Rac1 inhibition decreased clonal growth and promoted clonal conversion (new Fig 5). These experiments explain that radial distribution of actin filaments (holoclone-type actin organization) is essential for holoclone-colony dynamics and maintenance of growth capacity of holoclones, but not sufficient for restoration of growth capacity of paraclones. We have discussed this point in the "Discussion" section.

- Figure 5: The effect of Akt and Rac1 inhibition should be tested in long term treatment and assess the rate of clonal conversion.

A: According to your suggestion, we have provided new results that show that long-term treatment of Akt and Rac1 inhibitors affected the expression of differentiation markers (new Fig S8). We also developed a doxycycline-inducible gene silencing system by using a lentivirus carrying shRNA, because these inhibitors might have side effects in long-term treatment. shRNA experiments revealed that knockdown of Akt1 and Akt2 also reduced Rac1 phosphorylation (new Fig 5E), as shown in the inhibitor experiment (new Fig 5B). The RNAi experiments also demonstrated that knockdown of Rac1 strikingly decreased growth capacity of cultured keratinocytes, and accelerated clonal conversion (new Fig 5E-G), as Rac1 inhibitor did (new Fig 5A and 5D). These data strongly support our conclusion that Rac1 inhibition promotes clonal conversion.

Surprisingly, shRNA knockdown of Akt1 and Akt2 resulted in the decreased expression of Rac1 protein, and accelerated clonal conversion (new Fig 5E-G). Procedures of lentivirus infection and induction of non-targeted control shRNA by doxycycline treatment did not affect Rac1 expression, growth capacity, and rate of clonal conversion (new Fig 5E-G). An Akt inhibitor also decreased colony-forming efficiency (CFE) (new Fig 5D), as Akt1 and Akt2 shRNA did (new Fig 5G), but neither reduced Rac1 expression (new Fig 5B), nor accelerated clonal conversion (new Fig 5D). This result suggests the existence of kinase activity-independent regulation of Rac1 expression by Akt1/2 in keratinocytes. We have discussed this point in the "Discussion" section. This growth

inhibition by shRNA-knockdown made impossible to culture the keratinocytes successively and to measure the rate of clonal conversion by serial cultivation (new Fig 5G).

Minor comments

Higher magnification of the IF showing the actin organization should be provided.

A: According to your suggestion, we have provided new images of rhodamine-phalloidin staining with higher magnification (new Fig 2A, new Fig 4B, and new Fig S7B). We also performed a population and quantitative study on the percentage of angles of each actin filament against plasma membrane in the cells located at the periphery of growing and terminal colonies (new Fig 2A, new Fig 3D and 3H, new Fig 4B, and new Fig S7B), by measuring rhodamine-palloidin staining images. These data clearly indicated the orientation of actin filaments in cells of growing and terminal colonies in each experimental condition. The details of this study are described in "Material and Methods" of the text and Supporting Information Figure S4.

The manuscript is sometime difficult to read and would probably benefit from being checked and edited by a non-specialist to ensure readability and clarity for a broad audience.

A: According to your suggestion, we have described signalling pathways in more detail in the "Results" section and added the explanation as to why each experiment was performed. The manuscript has received critical reading by non-specialists to improve the readability.

For Referee #2

The manuscript by Namba and co-workers analyse the actin cytoskeleton organisation of primary keratinocytes. They propose that holo clones and paraclones can be differentiated by specific aspects of the actin filament structures, which leads to changes in colony behaviour, response to EGF and proliferative capacity of different clones. The manuscript is well written and figures of high quality.
However, this reviewer has not been persuaded by the argument or conclusions in the manuscript. The phenotypes observed can have alternative explanations that do not involve clone conversion. The data are thus over-interpreted and not supported by the results shown.

The actin cytoskeleton is very dynamic and can remodel very quickly in response to different stimuli. Importantly, actin organisation in keratinocytes is very variable, and thus without a population study on percentage of cells that show a particular organisation of actin, the data is not convincing. I understand that time lapse experiments are laborious, but the quantification and classification of cells that have a particular organization of the cytoskeleton can be done in fixed samples.

A: According to your suggestion, we have performed a population and quantitative study on the percentage of angles of each actin filament against the plasma membrane in the cells located at the periphery of growing and terminal colonies (new Fig 2A, new Fig 3D and 3H, new Fig 4B, and new Fig S7B), by measuring rhodamine-palloidin staining images. These data clearly indicated the orientation of actin filaments in cells of growing and terminal colonies in each experimental condition. The details of this experiment are described in the "Material and Methods" section and Supporting Information Figure S4.

Delineation of pathways is achieved by long term treatment with inhibitors that per se can clearly have toxic, non-specific effects in cells. First, these inhibitors are not specific to the proteins mentioned, and thus contribution of other proteins for the phenotype cannot be excluded (see papers by Bain et al 2003 and 2007). Confirmation of the results with RNAi is then very important to substantiate the conclusions.

A: According to your suggestion, we have developed a doxycycline-inducible gene silencing system by using a lentivirus carrying shRNA, and revealed that knockdown of Akt1 and Akt2 also reduced Rac1 phosphorylation (new Fig 5E), as shown in the inhibitor experiment (new Fig 5B). The RNAi experiments also demonstrated that knockdown of Rac1 strikingly decreased growth capacity of

cultured keratinocytes and accelerated clonal conversion (new Fig 5E-G), as Rac1 inhibitor did (new Fig 5A and 5D). These data strongly support our conclusion that Rac1 inhibition promotes clonal conversion.

Surprisingly, shRNA knockdown of Akt1 and Akt2 resulted in the decreased expression of Rac1 protein, and accelerated clonal conversion (new Fig 5E-G). Procedures of lentivirus infection and induction of non-targeted control shRNA by doxycycline treatment did not affect Rac1 expression, growth capacity, and rate of clonal conversion (new Fig 5E-G). An Akt inhibitor also decreased colony-forming efficiency (CFE) (new Fig 5D), as Akt1 and Akt2 shRNA did (new Fig 5G), but neither reduced Rac1 expression (new Fig 5B), nor accelerated clonal conversion (new Fig 5D). This result suggests the existence of kinase activity-independent regulation of Rac1 expression by Akt1/2 in keratinocytes. We have discussed this point in the "Discussion" section.

Second, if looked at face value, the same phenotypes on cytoskeletal organization shown for inhibition of Akt, PI3K, etc. in different cell types are now shown again in keratinocytes and proposed to regulate clone conversion. It could be that enlargement of colony area just reflects that (i) spreading of the keratinocytes in general, and (ii) the perturbed response of terminal colonies is because of a rewiring of signalling related to Rac signalling to spreading (rather than clone conversion).

A (i): Time-lapse imaging clearly demonstrated that EGF-induced expansion (enlargement) of colony area resulted from centrifugal migration of the peripheral cells with maintenance of cell-cell contact, and a flattening of the cells located at the centre of the colony (Supporting Information Movie S1). Therefore, EGF-induced colony expansion does not reflect only the spreading of the keratinocytes.

A (ii): Our results indicate that shrinking of terminal colonies is due to interaction of myosins with circumferential actin network through EGFR/ERK/MLCK signalling. This mechanism also underlies EGF-induced expansion of growing colonies with radial organization of actin filaments (new Fig 6A). The difference in actin filament organization between holoclones and paraclones is already determined by clonal conversion (new Fig 6B).

Analysis of MLC phosphorylation following treatment with different inhibitors is simplistic and contains flaws. For example some of the inhibitors used disassemble actin bundles and thus obviously there will be no phosphorylated MLC staining on bundles because the bundles are not there. This conclusion can only be made if pMLC is reduced without perturbation of the actin fibres.

A: As you mentioned, Y27632 (a ROCK inhibitor) disassembled actin filaments, and inhibited EGF-induced colony dynamics. However, U0126 and PD98059 (MEK inhibitors), and ML7 (a MLCK inhibitor) did not disturb actin filament network, but inhibited MLC phosphorylation and EGF-induced colony dynamics (new Fig1D and 1E, new Fig 2D, and new Fig S2B and S2D). In addition, MLC phosphorylation was detected even if the actin bundles were disorganized in the presence of blebbistatin (new Fig 3C and new Fig S2C).

Long-term treatment of rapamycin (3 days) did not disturb the actin filament network, but inhibited MLC phosphorylation and EGF-induced colony dynamics (new Fig 3F-H and new Fig S2E). These data strongly support our conclusion that EGF-induced colony dynamics (expansion of growing colonies and shrinking of terminal colonies) depends on MLC phosphorylation through EGFR/ERK/MLCK signalling.

Figure 2a- cells shown are not comparable. On top, the cell lies in the middle of the colony, surrounded by neighbouring cells, while in the bottom panel, cell is at the periphery of the colony, and thus able to protrude lamella.

A: Actually, cells on the top of old Fig 2A lied at the periphery of a growing colony. But irradiated 3T3 cells were near the periphery of the colony. It seemed that cells lying at the periphery of the growing colony were still surrounded by neighbouring keratinocytes, as you mentioned, and this will make readers confused. According to your suggestion, we have replaced old images with new images (new Fig 2A), so that reviewers and readers can clearly recognize keratinocytes located at the periphery of the colony.

Image used for control +EGF in figure 3C is the same used previously in Fig.2D.

A: We used the same image for control in old Fig 2D and 3C, because both experiments were done at the same day, under the same conditions. However, this will also make readers confused. According to your suggestion, we have replaced the control +EGF image (old Fig 3C) with another image that was also obtained at the same day, under the same conditions (new Fig 3C).

Authors should specify if de-lipidated BSA was used to add to EGF onto cells. If not, standard BSA contains lipids that can stimulate Rho and MLC phosphorylation.

A: We have used standard BSA (Sigma #A4919) for all experiments. This BSA is validated for human keratinocyte stem cell culture in our lab. The final concentration of BSA in culture medium was 0.003% and 0.001% in all short- and long-term experiments, respectively. According to your suggestion, we confirmed that addition of BSA (0.003%) did not change the level of phosphorylation of MLC in cultured keratinocytes (new Fig S2F).

For Referee #3

This paper would be greatly improved by clearer explanation of the experiments, the rationale for which is hard to follow without a good knowledge of the signalling pathways involved. While the actin results are of some interest, the observation of the role of Rac1 in human epidermal stem cells is the most important point. This may not be directly linked with the actin effect.

Primary cultures of human keratinocytes are important in regenerative medicine as this system is in routine use for ex vivo expansion for autologous transplantation in patients with burns. This manuscript examines the relationships between the actin cytoskeleton and clonogenic potential in primary cultures of human keratinocytes, in order to better define the events associated with a loss of stem cell self renewal that limits the expansion of cells in culture. The investigations on actin cytoskeleton in different colony types in this study lead to the identification of Rac1 as essential for sustaining the growth of stem cell derived colonies, a finding which extends previous observations in mice to the human system.

Abstract:
It would be worth explaining the role of cultured keratinocytes in therapy and the terms holoclone and paraclone more clearly as these would not be familiar to a non specialist reader. The link between EGF and RAC signalling could also be explained.

A: According to your suggestion, we explained the clinical uses of cultured human keratinocyte stem cells, and the terms "holoclone" and "paraclone" more clearly in the "Abstract". We also explained directly that EGF-induced colony dynamics was governed by Rac1.

Results:
The authors begin by demonstrating that terminally differentiating colonies shrink in response to EGF treatment whilst larger proliferating colonies expand. The expansion of larger colonies is blocked by MEK and MLCK inhibition but the shrinking of smaller colonies is refractory to these agents. It would be helpful to have a line or two explaining the EGFR/ERK/MLCK pathway and the rationale for using the inhibitors for readers not familiar with this aspect of signalling.

A: According to your suggestion, we have described EGFR/ERK/MLCK pathway in more detail in the "Results" section and drawn a schematic representation showing that the specific inhibitors blocked in this pathway (new Fig 1E and 1D).

Next the actin cytoskeleton is examined (Fig. 2) and differences in F actin organization observed between growing and differentiated colonies in the presence of EGF and/or inhibitors. The relationship between actin organization and colony type is shown to be independent of passage number (Fig S2).

In Figure 3 the effect of drugs that increase keratinocyte clonogenicity is examined. The ROCK inhibitor Y27632 is found to block the effects of EGF in both colony types. Short term treatment with rapamycin has little effect, whereas long term treatment, argued to act on mTORC2 blocks the effect of EGF on both colony types. The activity of these agents should be confirmed by Western blotting, as has been done for the other inhibitors, particularly to demonstrate the difference between short vs long term rapamycin treatment.

A: According to your suggestion, we have performed Western blotting experiments using antibodies against S6K1, Akt, MLC, and their phosphorylated forms, and confirmed that short-term rapamycin treatment inhibited mTORC1 activity (S6K1 phosphorylation), and that the long-term treatment inhibited mTORC1 and mTORC2 activity (Akt Ser473 phosphorylation), and MLC phosphorylation (Fig S2E).

In Figure 4 PI3K signalling is investigated, again using inhibitors. PI3K or Rac1 inhibition reorganises actin in both colony types, blocks the EGF response of growing colonies and augments the EGF effect on terminally differentiating colonies.

Figure 5 demonstrates that Rac1 inhibition potently inhibits colony growth, triggering the transition from actively growing to terminally differentiating colonies. Akt inhibition has a similar effect. Given that the important conclusion of this study is the link between Rac1 and clonogenicity, confirming the role of Rac1 with shRNA knockdown would strengthen the argument.

A: According to your suggestion, we have developed a doxycycline-inducible gene silencing system by using a lentivirus carrying shRNA, and revealed that knockdown of Akt1 and Akt2 also reduced Rac1 phosphorylation (new Fig 5E), as shown in the inhibitor experiment (new Fig 5B). The RNAi experiments also demonstrated that knockdown of Rac1 strikingly decreased growth capacity of cultured keratinocytes, and accelerated clonal conversion (new Fig 5E-G), as Rac1 inhibitor did (new Fig 5A and 5D). These data strongly support our conclusion that Rac1 inhibition promotes clonal conversion.

Surprisingly, shRNA knockdown of Akt1 and Akt2 resulted in the decreased expression of Rac1 protein, and accelerated clonal conversion (new Fig 5E-G). Procedure of lentivirus infection and induction of non-targeted control shRNA by doxycycline treatment did not affect Rac1 expression, growth capacity, and rate of clonal conversion (new Fig 5E-G). An Akt inhibitor also decreased colony-forming efficiency (CFE) (new Fig 5D), as Akt1 and Akt2 shRNA did (new Fig 5G), but neither reduced Rac1 expression (new Fig 5B), nor accelerated clonal conversion (new Fig 5D). This result suggests the existence of kinase activity-independent regulation of Rac1 expression by Akt1/2 in keratinocytes. We have discussed this point in the "Discussion" section.

In general the explanation as to why each experiment is performed could be greatly improved for readers unfamiliar with the pathways involved.

A: According to your suggestion, we have described signalling pathways in more detail in the "Results" section and added the explanation as to why each experiment was performed. The manuscript has received critical reading by non-specialists to improve the readability.

The study concludes "remodelling of actin filaments and inhibition of actomyosin contractility can impact stem cell behaviour through the modulation of clonal conversion". Whilst actin remodelling is associated with differentiation it is not clear that effects on the actin cytoskeleton itself underlie conversion.

A: Y27632 could not rescue a paraclone from its commitment to a terminal phenotype even if this inhibitor remodelled actin filament organization of paraclones to that of holoclones (new Fig 3D and Supporting Information Fig S6B). This means that remodelling of actin filaments from paraclone- to holoclone-type is not involved in decreased clonal conversion. However, remodelling of actin filaments from holoclone- to paraclone-type by Rac1 inhibition decreased clonal growth, and promoted clonal conversion (new Fig 5). These experiments explain that radial distribution of actin filaments (holoclone-type actin organization) is essential for holoclone-colony dynamics and

maintenance of growth capacity of holoclones, but not sufficient for restoration of growth capacity of paraclones. We have discussed this point in the "Discussion" section.

2nd Editorial Decision 27 January 2013

Thank you for the submission of the revised version of your manuscript to EMBO Molecular Medicine. We have now heard back from the three Reviewers whom we asked to re-evaluate your manuscript.

You will see that all three Reviewers support publication of your work. However, two Reviewers have some remaining concerns that require your action. For this reason, publication of the paper cannot be considered at this stage.

I feel that there is no need to discuss each point in detail as they are clearly stated. In brief, Reviewer 1 asks you to show the experiments demonstrating that the remodeling of actin filaments was not sufficient to reverse clonal conversion as reported in the Discussion. Reviewer 2 points to several issues that need to be explained/discussed. Firstly, s/he maintains that to state that PI3K/Akt stimulation during serial cloning leads to decreased Rac1 activity is misrepresentative and should be clarified. In addition s/he questions the data reported in Fig.5d. Reviewer 2 also mentions two clear instances of over-statement that need to be corrected.

As you know, we would normally not allow a second revision. I am prepared in this case, however, to give you the opportunity to improve your manuscript further, with the understanding that the Reviewers' concerns must be fully and carefully addressed. I am prepared to make an editorial decision on your revised version, but please provide a detailed point-by-point response and the clear indication of where the required modifications have been incorporated into the new manuscript. Acceptance or rejection of the manuscript will depend on the completeness of your responses included in the next, final version of the manuscript.

I look forward to receiving your revised manuscript as soon as possible.

***** Reviewer's comments *****

Referee #1 (General Remarks):

In this revised version, the authors responded to our comments. The brief explanations on the molecular mechanisms of the signaling pathways made the manuscript more pleasant to read and easily understandable to non-molecular biology specialist. Adding the RNAi experiments confirms previous results using pharmacologic inhibitors and with increased specificity.

Specific comment:

The authors mentioned in their response that they tested the effects of 3 molecules inhibiting the polymerization of actin filament (cytochalasin D, mycalolide B and bistheonelide A) on paraclones to assess if this can revert paraclones to holocolones. The results were negative. They conclude that remodeling of acting filaments was not sufficient to reverse clonal conversion. This point is discussed in the Discussion. Nevertheless, this is a key point and these experiments need to be shown in the paper.

Referee #2 (General Remarks):

The revised version of the manuscript is substantially improved. The authors toned down their claims on the importance of actin as the sole determinant of clonal conversion and the text is more focused and clearer. At some places there are still conclusions implying causal relationship, which

has not been formally shown. Addition of the quantification of actin organisation profiles also strengthened their data, which now show convincingly the population distribution of the different actin structures. However, a point that requires revision is that the distinct actin organisation most likely enables differential responses to EGF and differentiation signals, rather than the other way around. Prevalence of different actin structures may be a read-out for ways to rewire distinct signalling pathways and thus cellular responses.

The statement in page 16 that PI3K/Akt stimulation during serial cloning has the consequence to decrease Rac1 activity is misleading. To my knowledge, PI3K is usually required for Rac1 activation in other model systems and as shown in Fig.4a. Regarding Akt, it is probable that interference with Rac1 signalling may relate to the strong reduction in Rac1 protein levels as off-target effect of Akt siRNA (Fig.5e). This possibility does not detract from the proposed kinase-independent effects and should be added to the discussion.

One piece of data that is not properly discussed or explained is Fig.5d. PI3K inhibition, in spite of strong inactivation of Rac1 and reduced Rac1 phosphorylation, does not decrease colony forming. Is this image representative? If so, some explanations are in order on how this fits with the model.

Fig.6b diagram shows mTORC2 modulation of Akt during clonal conversion. This has not been formally shown in this manuscript and should be removed.

Page 15 1st paragraph. The phrase "our data strongly suggest that EGF-induced colony dynamics of actin filaments governs colonial growth and conversion" is an over-interpretation of the data and should be toned down.

Referee #3 (General Remarks):

The authors have addressed the key points highlighted in the first review. The clarity of the manuscript has been improved and the additional RNAi experiments strengthen the argument significantly.

2nd Revision - authors' response 13 February 2013

Referee #1

In this revised version, the authors responded to our comments. The brief explanations on the molecular mechanisms of the signalling pathways made the manuscript more pleasant to read and easily understandable to non-molecular biology specialist. Adding the RNAi experiments confirms previous results using pharmacologic inhibitors and with increased specificity.

Specific comment:

The authors mentioned in their response that they tested the effects of 3 molecules inhibiting the polymerization of actin filament (cytochalasin D, mycalolide B and bistheonelide A) on paraclones to assess if this can revert paraclones to holocolones. The results were negative. They conclude that remodelling of acting filaments was not sufficient to reverse clonal conversion. This point is discussed in the Discussion. Nevertheless, this is a key point and these experiments need to be shown in the paper.

We have added the requested experiments using three small molecules, including an actin polymerization inhibitor (cytochalasin D), and two compounds that induce depolymerisation of actin filaments (mycalolide B and bistheonelide A) into the Supplemental Information (Fig S7). All three molecules did not enhance colony-forming efficiency and clonal growth of keratinocytes, and this result clearly indicated that these molecules could not convert paraclones into holoclones. We have now mentioned this result in the “Results” (p10, line 2-7) and discussed in the “Discussion” (p15, line 16-18) of the text.

Referee #2

The revised version of the manuscript is substantially improved. The authors toned down their claims on the importance of actin as the sole determinant of clonal conversion and the text is more focused and clearer. At some places there are still conclusions implying causal relationship, which has not been formally shown. Addition of the quantification of actin organisation profiles also strengthened their data, which now show convincingly the population distribution of the different actin structures. However, a point that requires revision is that the distinct actin organisation most likely enables differential responses to EGF and differentiation signals, rather than the other way around. Prevalence of different actin structures may be a read-out for ways to rewire distinct signalling pathways and thus cellular responses.

The statement in page 16 that PI3K/Akt stimulation during serial cloning has the consequence to decrease Rac1 activity is misleading. To my knowledge, PI3K is usually required for Rac1 activation in other model systems and as shown in Fig.4a. Regarding Akt, it is probable that interference with Rac1 signalling may relate to the strong reduction in Rac1 protein levels as off-target effect of Akt siRNA (Fig.5e). This possibility does not detract from the proposed kinase-independent effects and should be added to the discussion.

We have first mentioned this point in "Results" in the text (p11, line 14-16) and shown the result in Fig. 4A. In our model, PI3K activity is constant and required for both Rac1 and Akt activity, but Akt activity becomes predominant during serial cultivation. As the reviewer rightly pointed it out, our previous statement could mislead readers. We have thus replaced our previous statement (page 16) by "Our findings indicate that activation of Akt signalling becomes predominant during serial cultivation, with the consequence of decreasing Rac1 activity" (p16, line 23-25; discussion).

Concerning Akt RNAi experiments, the interference with Rac1 signalling by shRNA knockdown of Akt 1 and Akt2 was not examined in this manuscript. As mentioned by the reviewer, the possibility that the interference with Rac1 signalling by Akt1/2 knockdown results in decreased Rac1 protein levels cannot be excluded. We have thus added the following sentence "It is likely that Akt1/2 is required for Rac1 signalling and that shRNA knockdown of Akt1/2 interferes with Rac1 signalling and decreases Rac1 protein levels consequently" (p17, line 11-13 ; discussion).

One piece of data that is not properly discussed or explained is Fig.5d. PI3K inhibition, in spite of strong inactivation of Rac1 and reduced Rac1 phosphorylation, does not decrease colony forming. Is this image representative? If so, some explanations are in order on how this fits with the model.

PI3K inhibition results in Rac1 inactivation and reduced Rac1 phosphorylation. Importantly, Rac1 phosphorylation inhibits Rac1-GTP binding and decreases Rac1 activity (Kwon et al., J Biol Chem 275, 423-428, 2002), as described (p12, line 20-22; results). Therefore, PI3K inhibition causes both Rac1 inactivation and activation, suggesting that the level of Rac1 activity is stable even when a PI3K inhibitor is present and/or that PI3K inhibition does not affect a balance between Rac1 and Akt activity. This notion fits our model and explains why the presence of a PI3K inhibitor did not reduce colony-forming efficiency. We have discussed this point in morer details and explained why PI3K inhibition does not decrease colony-forming efficiency in the "Discussion" of the text (p17, line 3-7 ; discussion).

Fig.6b diagram shows mTORC2 modulation of Akt during clonal conversion. This has not been formally shown in this manuscript and should be removed.

We have removed "mTORC2" from Fig.6B and the related sentences.

Page 15 1st paragraph. The phrase "our data strongly suggest that EGF-induced colony dynamics of actin filaments governs colonial growth and conversion" is an over-interpretation of the data and should be toned down.

We agree that the data from Rheinwald and Green (Nature 265, 421-424, 1977), Barrandon and Green (Cell 50, 1131-1137, 1987), and the present study are suggestive but not definitive concerning the connection between EGF and clonal conversion. We have changed this phrase into “These data suggest that EGF-induced colony dynamics defined by actin filament organization is involved in clonal growth and conversion” (p15, line 13-14).
