# Supplementary material for: Actin filament dynamics impacts keratinocyte stem cell maintenance
Source: EMBO Mol Med. 2013 Apr 2;5(4):640–53. doi: 10.1002/emmm.201201839 (PMC3628097; doi:10.1002/emmm.201201839)

Supporting Information

Actin Filament Dynamics Impacts Keratinocyte Stem Cell Maintainance

Daisuke Nanba[1,2,3,4], Fujio Toki[1,2,3,4], Natsuki Matsushita[4], Sachi Matsushita[4], Shigeki Higashiyama[4,5], and Yann Barrandon[1,2]

[1]Laboratory of Stem Cell Dynamics, Ecole Polytechnique Fédérale de Lausanne (EPFL), CH-1015 Lausanne, Switzerland, [2]Department of Experimental Surgery, Centre Hospitalier Universitaire Vaudois (CHUV), CH-1011 Lausanne, Switzerland, and [3]Senior Research Fellow Center, [4]Proteo-Medicine Research Center (ProMRes), and [5]Department of Biochemistry and Molecular Genetics, Graduate School of Medicine, Ehime University, Shitsukawa, Toon, Ehime 791-0295, Japan.

Table of contents

Legends for Supporting Information Figure S1-S9 (page 2-7)

Legends for Supporting Information Movie S1 and S2 (page 8)

Supporting Information Table S1-S4 (page 9-12)

Supporting Information Figure S1-S10 (page 13-22)

Supporting Information Figure S1: (A) Macroscopic appearance of rhodamine B stained colonies of human epidermal keratinocytes after 12 days of culture, according to Rheinwald and Green (1975). Scale bar is 10 mm. (B) The area of 20 growing and 16 terminal colonies shown in (A) was measured with MetaMorpho (Molecular Devices) using a graphic tablet (Wacom). Size distribution shows that terminal colonies are extremely small. (Top) Phase-contrast microscopic images of growing and terminal colonies after 6 days of cultivation without EGF, and enlarged photographs of a growing and a terminal colony at day 12 as shown in (A). Scale bars: 50 μm and 1 mm respectively.

Supporting Information Figure S2: (A) Immunofluorescence with ERK1/2 and phosphorylated ERK1/2 (pERK1/2) antibodies in growing and terminal keratinocyte colonies. After 5 min, EGF strongly induced ERK1/2 phosphorylation in both growing and terminal colonies, while it was inhibited by U0126 (10 μM). Scale bar: 50 μm. (B) Relative increase in colony area after addition of EGF in presence of DMSO (vehicle), and PD98059 (50 μM). (C) Western blotting of lysates from keratinocytes exposed to various inhibitors. After 30 min, EGF induced phosphorylation of EGFR, ERK1/2 and MLC in keratinocytes. (D) Growing and terminal colonies were immunostained with a fluorescent phosphorylated MLC (pMLC) antibody. After 30 min, EGF induced strong MLC phosphorylation in both growing and terminal colonies, while it was inhibited by U0126 or ML7. Scale bar: 50 μm. (E) Western blotting of lysates from keratinocytes exposed to rapamycin for 1 hour or 3 days. After 30 min, EGF induced phosphorylation of ERK1/2, S6K1, Akt and MLC in keratinocytes. However, long-, but not short-, term treatment of keratinocytes with rapamycin decreased EGF-induced MLC

phosphorylation. We confirmed that short-term rapamycin treatment inhibited mTORC1 activity (S6K1 phosphorylation), and that the long-term treatment inhibited both mTORC1 and mTORC2 activity (Akt Ser473 phosphorylation). (F) Western blotting of lysates from keratinocytes exposed to BSA (vehicle) or EGF for 30 min. We confirmed that addition of BSA solution did not change the level of phosphorylation of MLC in cultured keratinocytes.

Supporting Information Figure S3: (A) Phase-contrast images of growing colonies of human epidermal keratinocytes. DMSO (vehicle) or U0126 (10 μM) were added to a 6 day-old growing colony, and the colony was cultured for 2 more days in the presence or absence of EGF. Photographs were taken at each time point (0, 24, and 48 hrs after EGF addition). Colony edges are outlined with white dots. U0126 inhibits EGF-induced expansion of a growing keratinocyte colony. Scale bar: 100 μm. (B) Relative increase in area of a growing colony area after EGF addition in the presence of DMSO or U0126. The values (mean ± S.D.) were determined from results obtained from at least 5 colonies. BSA solution was added instead of EGF solution in no EGF condition. (C) Immunofluorescence of a growing colony of keratinocytes with antibodies against involucrin (INV), keratin 1 (K1), transglutaminase 1 (TG1), and desmoglein 1 (DSG1) in presence of DMSO or U0126 for 48 hrs. Colonies were also stained with Hoechst 33258 to visualize DNA. Colony edges are outlined with white lines. U0126 increased expression of INV, K1, TG1, and DSG1, even when EGF was added to the culture. Scale bar: 100 μm. (D) Quantitative RT-PCR analysis for mRNA of *INV*, *K1,* and *keratin 10* (*K10*) in total RNA isolated from keratinocytes cultured for 48 hrs in presence of DMSO or U0126. A MEK inhibitor U0126 increased the amount of mRNA

of *INV*, *K1*, and *K10*.

Supporting Information Figure S4: A schematic representation of measurement of angles of actin filaments in cells of keratinocyte colonies. An angle of a single actin filament against the plasma membrane in a cell localized at the periphery of a keratinocyte colony was defined as an acute crossed-axes angle ($0^{o} \leq \theta \leq 90^{o}$) between a line (white in left panel) linking cell-cell contact points at colony edge, and a line (yellow in left panel) along a well-defined actin filament in a cell.

Supporting Information Figure S5: (A) Phase-contrast images and relative increase in colony area of human epidermal keratinocyte clones (#01 and #14) after addition of EGF. Scale bar: 50 μm. (B) Growing and terminal colonies initiated by the progeny of the keratinocyte clones (#01 and #14) were stained with rhodamine-phalloidin (left), and a pAkt antibody (right). Scale bars: 20 (left) and 50 (right) μm.

Supporting Information Figure S6: (A) Phase-contrast images of growing and terminal keratinocyte colonies after addition of EGF, and in presence of Y27632 (10 μM). Scale bar: 50 μm. (B) Y27632 and EGF (10 ng/ml) were added to a 4 day-old growing and terminal colonies, and cells were cultured for 4 more days before Y27632, but not EGF, was removed when the medium was changed. Colonies were fixed 4 days later and stained with rhodamine B. Center and right panels scale bars: 100 μm and 1 mm respectively. (C and D) Phase-contrast images of growing and terminal keratinocyte colonies after addition of EGF. Rapamycin (100 nM) was added for 1 hour (C) or 3 days (D) before EGF addition. Scale bar: 50 μm.

Supporting Information Figure S7: The effects of different concentrations of an actin polymerization inhibitor (cytochalasin D) and two compounds that induces depolymerization of actin filaments (mycalolide B, and bistheonelide A) on the growth capacity of human keratinocytes. Keratinocytes were seeded at high (A) (600 cells/well in 12 well-plate) or low (B) (100 cells/60 mm dish) density and cultured in the presence of these molecules. Cells were treated with each molecule until 4 days of cultivation, and cultured for 8 more days without inhibitors before the colonies were fixed and stained with rhodamine B. In B, Y27632 was used as positive control. Scale bars: 10 mm.

Supporting Information Figure S8: (A) Human epidermal keratinocytes were cultured in presence of wortmannin (100 nM) or Akt inhibitor (10 μM), proteins were extracted, and Western blotting was performed to detect Akt, pAkt, ERK1/2 and pERK1/2. (B) Actin filaments were visualized by rhodamine-phalloidin staining in cells localized at the periphery of growing and terminal colonies cultured for 6 days without EGF, but in presence of DMSO (vehicle), wortmannin, or Akt inhibitor. Upper panel: Distribution of angles of actin filaments against plasma membrane in cells localized at the periphery of growing and terminal colonies without EGF in presence of DMSO, wortmannin, or Akt inhibitor for 1 hour. Lower panel: Images of rhodamine-phalloidin staining of cells. Scale bar: 5 μm. (C and D) Phase-contrast images of growing and terminal colonies after addition of EGF in presence of wortmannin (C) or Akt inhibitor (D). Scale bar: 50 μm. (E and F) Relative increase in colony area of growing and terminal colonies after addition of EGF in presence of wortmannin (E) or Akt inhibitor (F).

Supporting Information Figure S9: (A) Quantitative RT-PCR analysis for mRNA of *INV, K1, K10, ΔNp63, Keratin 14 (K14)* in total RNA isolated from keratinocytes cultured for 3 days, in presence of different inhibitors. LY303511 (10 μM), LY294002 (10 μM), NSC23766 (50 μM), Akt inhibitor (10 μM), and rapamycin (100 nM) were added after 4 days of cultivation. (B) Western blotting of INV, TG1, DSG1, ΔNp63, K14, and GAPDH from keratinocytes cultured in the conditions described in (A). LY294002, Akt inhibitor, and rapamycin markedly decreased INV, TG1, and DSG1 expression. (C) Immunofluorescence of a growing colony of keratinocytes with antibodies against INV, K1, TG1, and DSG1 in presence of different inhibitors for 3 days. Colonies were also stained with Hoechst 33258 to visualize DNA. Colony edges are outlined with white lines. LY294002, Akt inhibitor, and rapamycin decreased expression of these differentiation markers. Note that NSC23766 increased INV, TG1, and DSG1 expression in growing keratinocyte colonies. Scale bar: 100 μm. (D) Macroscopic appearance of rhodamine B stained colonies of human epidermal keratinocytes after 12 days of culture. Each signaling inhibitor was added after 4 days of cultivation. Cells were cultured for 8 more days before the colonies were photographed. The number of colonies (mean ± S.D.) is indicated in each column. Scale bar: 10 mm.

Supporting Information Figure S10: (A) Structure of doxycycline-inducible miR-type shRNA lentiviral vector. (B-D) Lentiviral vectors carrying transgene cassettes of miR-typed shRNA sequences (#1 to #4) targeted to *Rac1* (B), *Akt1* (C), and *Akt2* (D) under the control of doxycyclin-inducible promoter were infected into HaCaT keratinocytes. Four days later, shRNA expression was induced by doxycycline treatment, and HaCaT cells were maintained for 3 more days, to be then lysed and analyzed the

expression of Rac1 (B), Akt (C), and Akt2 (D) protein by Western blotting.

Supporting Information Movie S1

Time-lapse imaging of a growing colony of human epidermal keratinocytes. Images were shot at 5 min intervals for 180 min after addition of EGF.

Supporting Information Movie S2

Time-lapse imaging of a terminal colony of human epidermal keratinocytes. Images were shot at 5 min intervals for 180 min after addition of EGF.

Supporting Information Table S1

List of pharmacological inhibitors

Name	Target	[C]	Manufacturer (catalog#)
U0126	MEK1/2	10 µM	Cell Signaling (9903)
PD98059	MEK1/2	50 µM	Cell Signaling (9900)
ML7	MLCK	25 µM	Calbiochem (475880)
Y27632	ROCK	10 µM	Calbiochem (688000)
(-)-Blebbistatin	Myosin	50 µM	Sigma-Aldrich (B0560)
LY303511	-	10 µM	Calbiochem (440203)
LY294002	PI3K	10 µM	Alexis biochemicals (270-038-M001)
Wortmannin	PI3K	100 nM	Cell Signaling (9951)
NSC23766	Rac1	50 µM	Calbiochem (553502)
Akt inhibitor VIII	Akt	10 µM	Calbiochem (124017)
Rapamycin	mTORC1	100 nM	Calbiochem (553211)
Cytochalasin D	Actin	-	Wako (599-03101)
Mycalolide B	Actin	-	Wako (132-12081)
Bistheonelide A	Actin	-	Wako (026-15391)

Supporting Information Table S2

List of antibodies for immunofluorescence microscopy and Western blotting

Antibody	Type	Manufacturer (catalog#)
Involucrin (SY5)	mouse monoclonal	gift from Dr. Watt
Transglutaminase1 (BC1)	mouse monoclonal	gift from Dr. Watt
Keratin1	rabbit polyclonal	Covance (PRB-149P)
p63	mouse monoclonal	Dako (M7247)
Cleaved notch 1 (Val1744)	rabbit polyclonal	Cell Signaling (2421)
Keratin 14	mouse monoclonal	Sigma-Aldrich (C8791)
EGFR	mouse monoclonal	BD Biosciences (610016)
Phospho-EGFR (Tyr845)	rabbit polyclonal	Upstate (07-820)
E-cadherin	mouse monoclonal	BD Biosciences (610404)
Desmoglein 1	rabbit polyclonal	Santa Cruze (SC-20114)
ERK1/2	rabbit monoclonal	Cell Signaling (4695)
Phospho-ERK1/2 (Thr202/Thr204)	rabbit monoclonal	Cell Signaling (9101)
MLC	rabbit polyclonal	Cell Signaling (3672)
Phospho-MLC (Ser19)	mouse monoclonal	Cell Signaling (3675)
Akt	rabbit polyclonal	Cell Signaling (9272)
Phospho-AKT (Ser473)	rabbit monoclonal	Cell Signaling (4058)
Akt1	rabbit monoclonal	Cell Signaling (2938)
Akt2	rabbit monoclonal	Cell Signaling (3063)
S6K1	rabbit polyclonal	Cell Signaling (9202)
Phospho-S6K1 (Thr389)	mouse monoclonal	Cell Signaling (4058)
Rac1	mouse monoclonal	Millipore (05-389)
Phospho-Rac1 (Ser71)	rabbit polyclonal	Cell Signaling (2461)

Supporting Information Table S3

List of primers for quantitative RT-PCR

Name	Sequence
human involucrin forward	AGGTCCAAGACATTCAAC
human involucrin reverse	CCCTTGTATGAGACGAT
human keratin 1 forward	GCCCTACTTTGAGTCAT
human keratin 1 reverse	GTCGAGACTGCGGTTG
human keratin 10 forward	TCAGATCGACAATGCC
human keratin 10 reverse	ACCAGTGGACACATTT
human keratin 14 forward	CTTCCGCACCAAGTAT
human keratin 14 reverse	ATACTGGTCACGCATC
human ΔNp63 forward	AGACTCAATTTAGTGAGC
human ΔNp63 reverse	CTGGGTAGTCGGTGTT
human TBP1 forward	ATAATCCCAAGCGGTT
human TBP1 reverse	ACTTTAGCACCTGTTAATAC

Supporting Information Table S4

List of sequences of miR-typed shRNA

Target	21nt target sequence	
RAC1 (NM_006908)	#3	CTAGTGGGAACTAAACTTGAT
AKT1 (NM_005163)	#2	CGGAGACTGACACCAGGTATT
AKT2 (NM_001626)	#4	CTATCTCAAACTCCTTGGCAA

Nanba et al., Figure S1

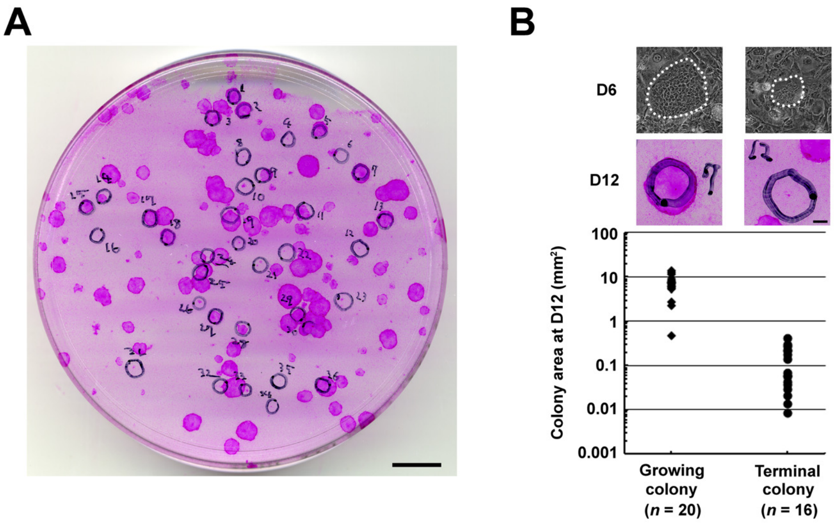

Nanba et al., Figure S2

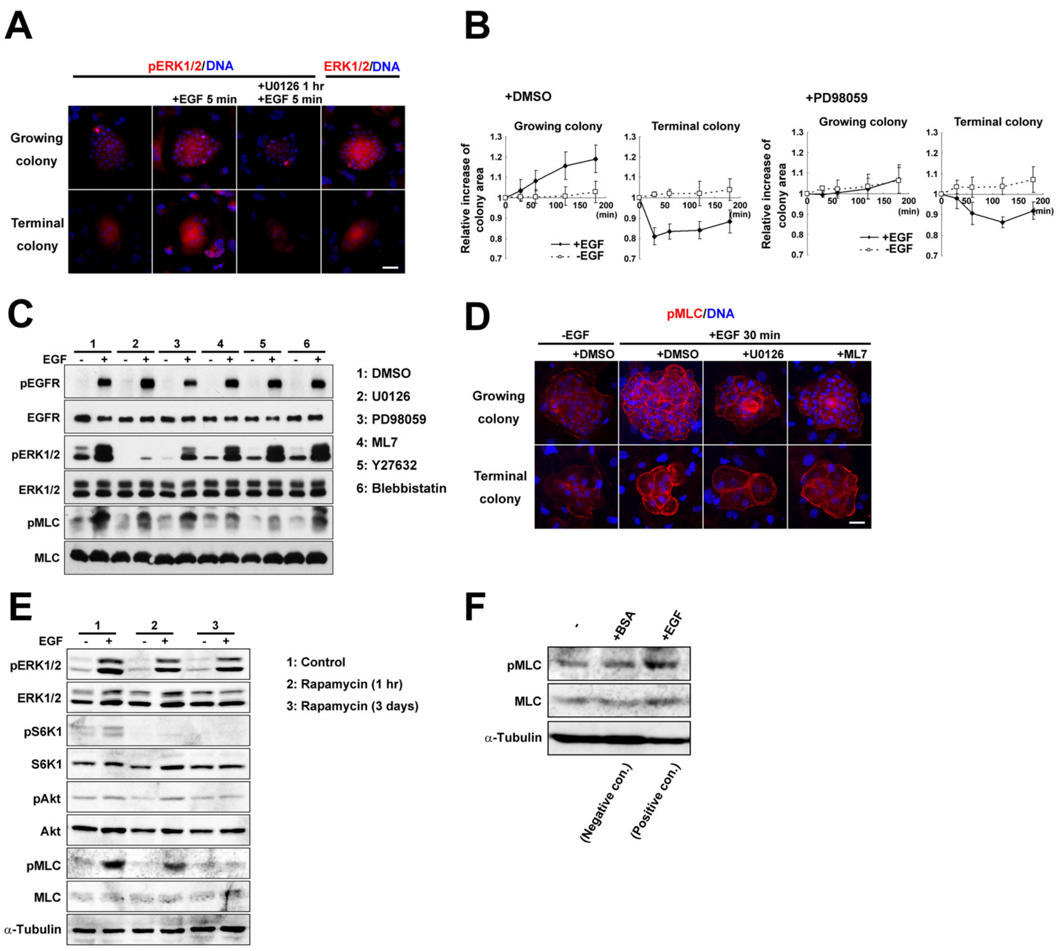

Nanba et al., Figure S3

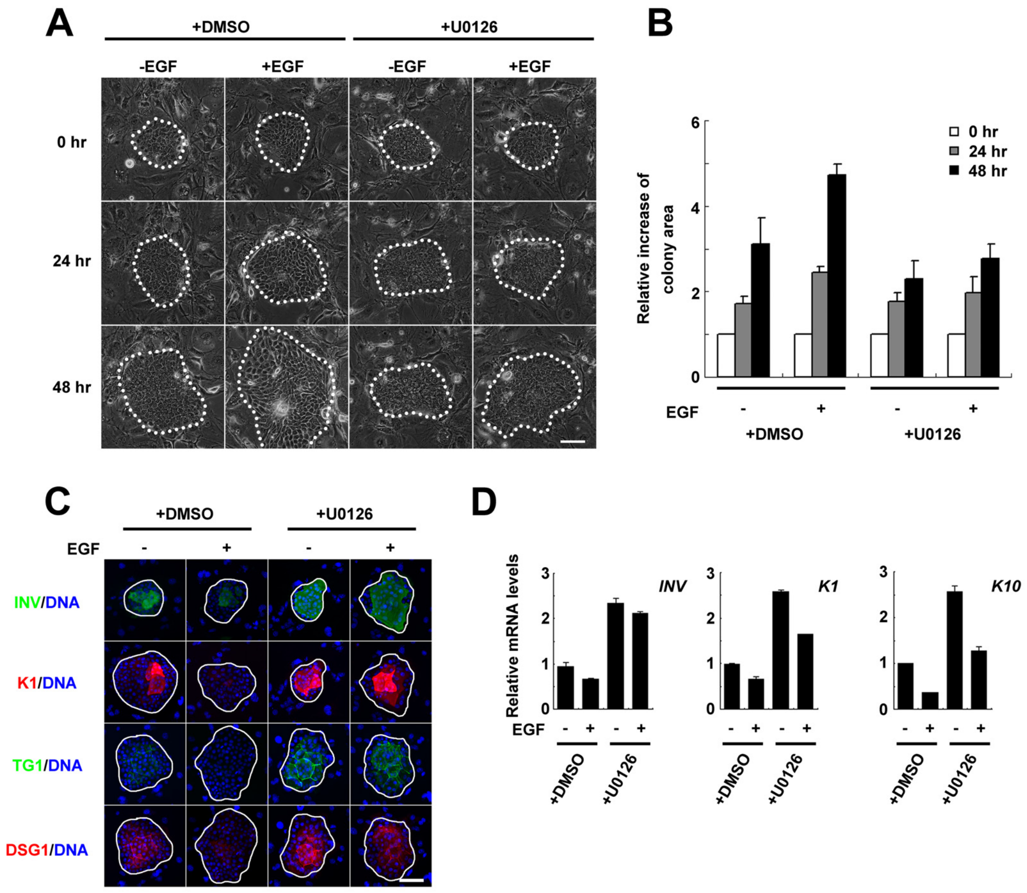

Nanba et al., Figure S4

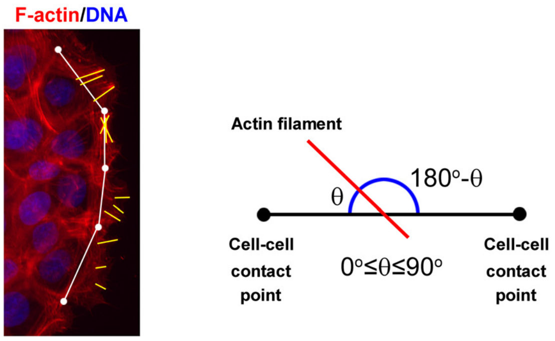

Nanba et al., Figure S5

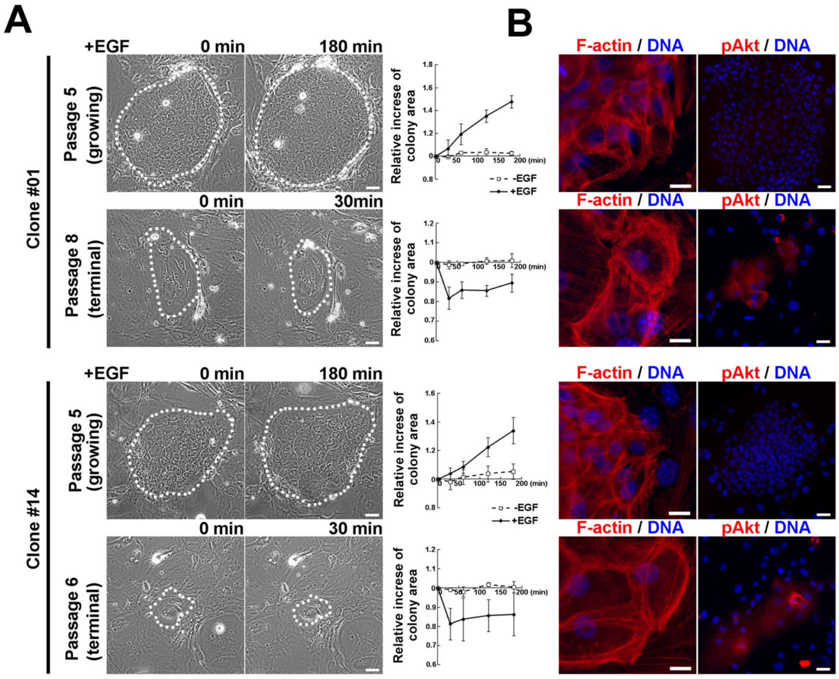

Nanba et al., Figure S6

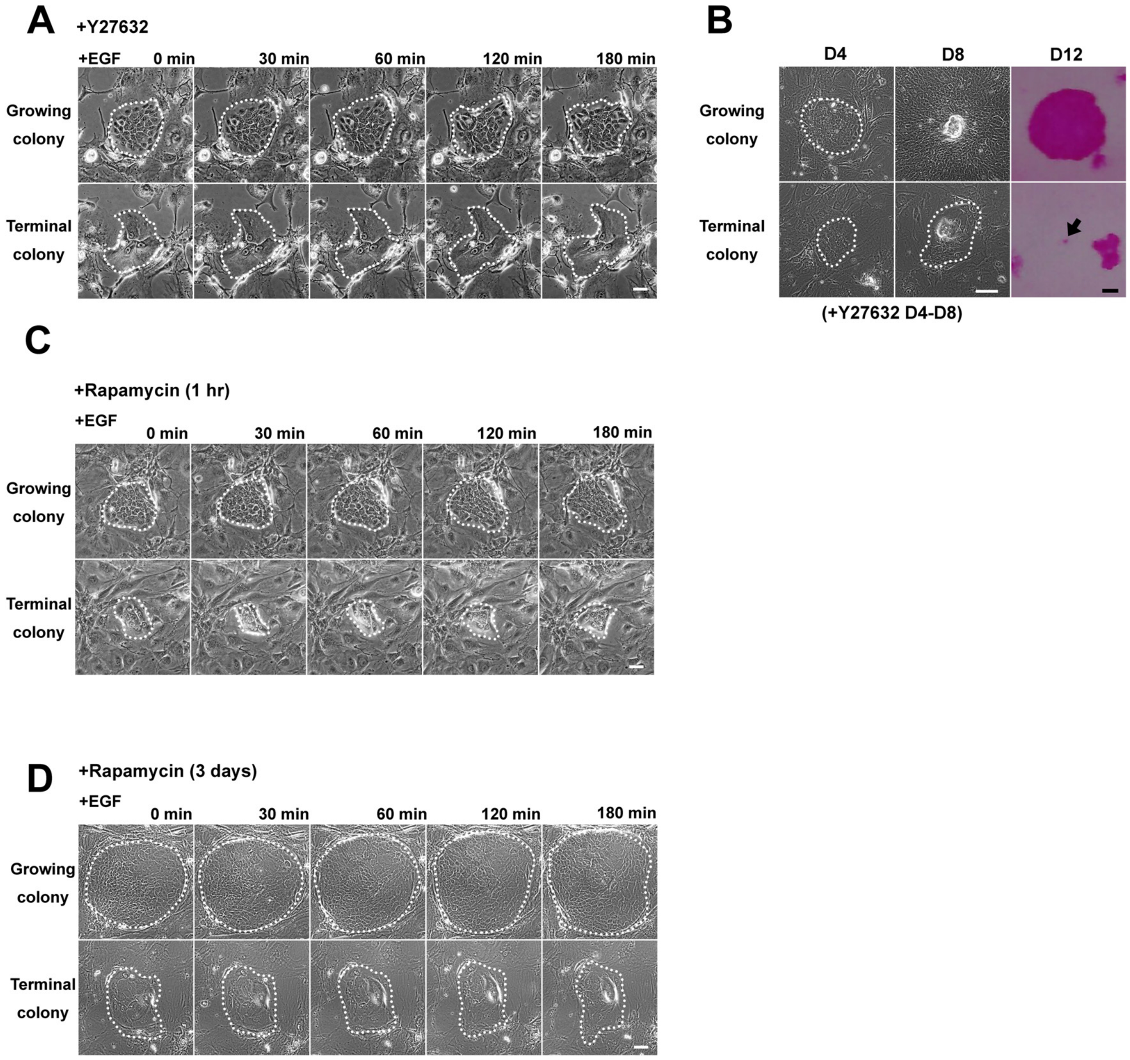

Nanba et al., Figure S7

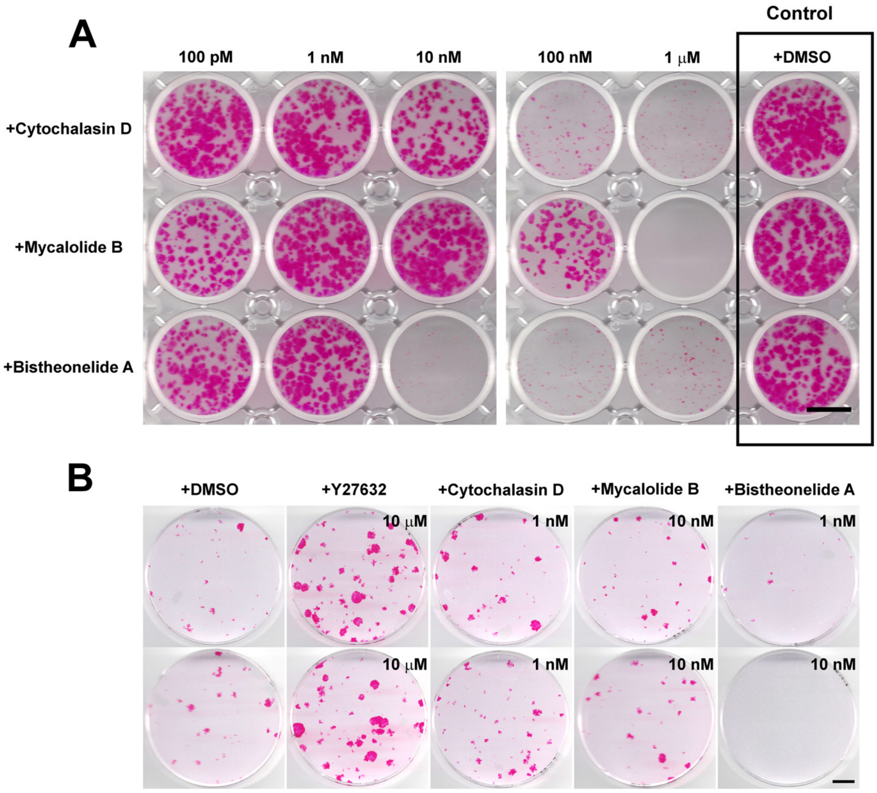

Nanba et al., Figure S8

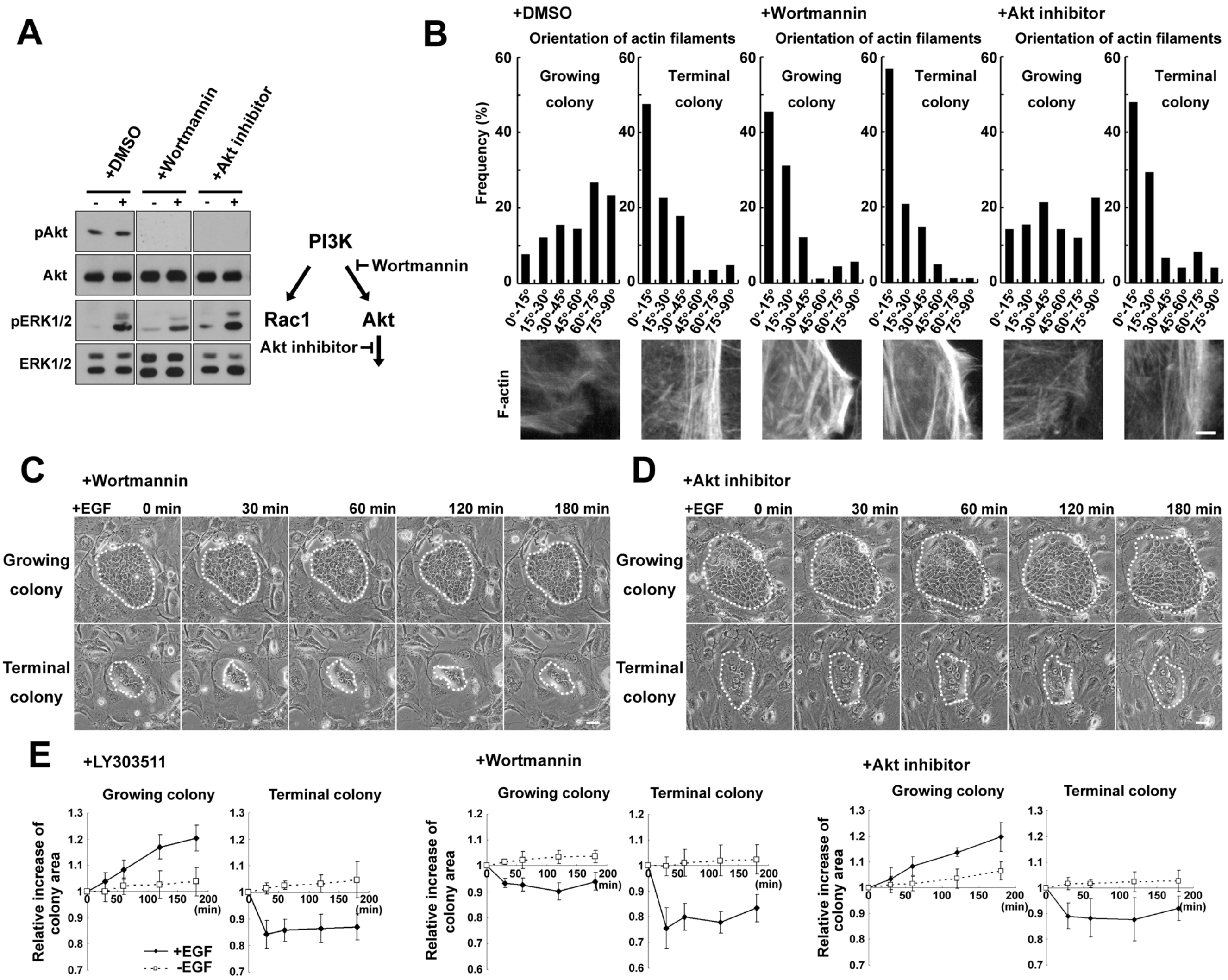

Nanba et al., Figure S9

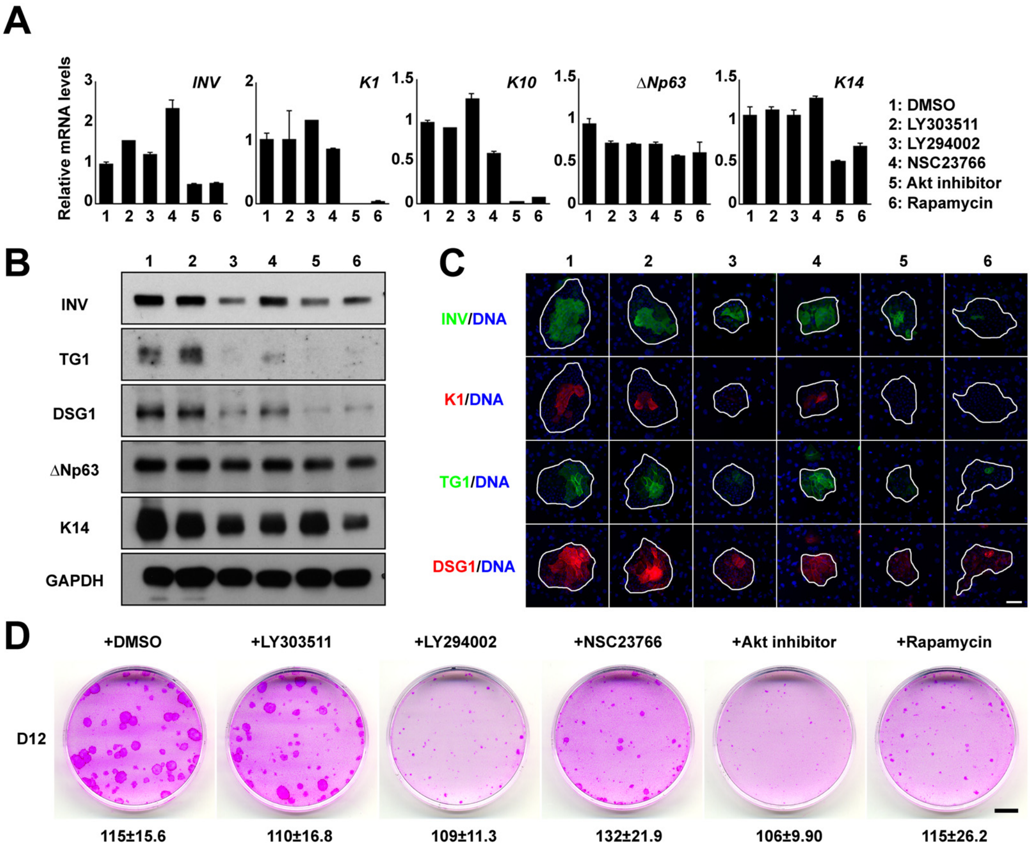

Nanba et al., Figure S10

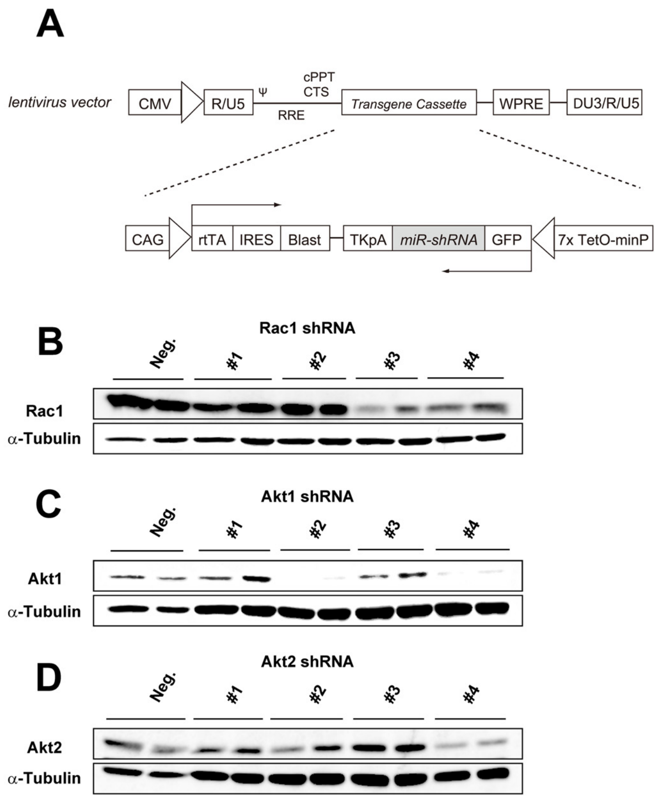

Supplement: Supplementary file 4 [file emmm0005-0640-sd4.pdf]
